# Influence of atlas-choice on age and time effects in large-scale brain networks in the context of healthy aging

**DOI:** 10.1162/imag_a_00127

**Published:** 2024-04-08

**Authors:** Pascal Frédéric Deschwanden, Alba López Piñeiro, Isabel Hotz, Brigitta Malagurski, Susan Mérillat, Lutz Jäncke

**Affiliations:** University Research Priority Program “Dynamics of Healthy Aging”, University of Zurich, Zurich, Switzerland; Healthy Longevity Center, University of Zurich, Zurich, Switzerland

**Keywords:** brain networks, resting-state fMRI, brain atlases, healthy aging, longitudinal study

## Abstract

*Introduction:*There is accumulating cross-sectional evidence of decreased within-network resting-state functional connectivity (RSFC) and increased between-network RSFC when comparing older to younger samples, but results from longitudinal studies with healthy aging samples are sparse and less consistent. Some of the variability might occur due to differences in network definition and the fact that most atlases were trained on young adult samples. Applying these atlases to older cohorts implies the generalizability of network definitions to older individuals. However, because age is linked to a less segregated network architecture, this assumption might not be valid. To account for this, the Atlas55+ (A55) was recently published. The A55 was trained on a sample of people over the age of 55, making the network solutions suitable for studies on the aging process. Here, we want to compare the A55 to the popular Yeo-Krienen atlas to investigate whether and to what extent differences in network definition influence longitudinal changes of RSFC. For this purpose, the following networks were investigated: the occipital network (ON, “visual network”), the pericentral network (PN, “somatomotor network”), the medial frontoparietal network (M-FPN, “default network”), the lateral frontoparietal network (L-FPN, “control network”), and the midcingulo-insular network (M-CIN, “salience network”).

*Methods:*Analyses were performed using longitudinal data from cognitively healthy older adults (*N*= 228, mean age at baseline = 70.8 years) with five measurement points over 7 years. To define the five networks, we used different variants of the two atlases. The spatial overlap of the networks was quantified using the dice similarity coefficient (DSC). RSFC trajectories within networks were estimated with latent growth curve models. Models of varying complexity were calculated, ranging from a linear model without interindividual variability in intercept and slope to a quadratic model with variability in intercept and slope. In addition, regressions were calculated in the models to explain the potential variance in the latent factors by baseline age, sex, and education. Finally, the regional homogeneity and the silhouette coefficient were computed, and the spin test and Wilcoxon-Mann-Whitney test were used to evaluate how well the atlases fit the data.

*Results:*Median DSC across all comparisons was 0.67 (range: 0.20–0.93). The spatial overlap was higher for primary processing networks in comparison to higher-order networks and for intra-atlas comparisons versus inter-atlas comparisons. Three networks (ON, PN, M-FPN) showed convergent shapes of trajectories (linear vs. quadratic), whereas the other two networks (L-FPN, M-CIN) showed differences in change over time depending on the atlas used. The 95% confidence intervals of the estimated time and age effects overlapped in most cases, so that differences were mainly evident regarding the*p*-value. The evaluation of the fit of the atlases to the data indicates that the Yeo-Krienen atlas is more suitable for our dataset, although it was not trained on a sample of older individuals.

*Conclusions:*The atlas choice affects the estimated average RSFC in some networks, which highlights the importance of this methodological decision for future studies and calls for careful interpretation of already published results. Ultimately, there is no standard about how to operationalize networks. However, future studies may use and compare multiple atlases to assess the impact of network definition on outcomes. Ideally, the fit of the atlases to the data should be assessed, and heuristics such as “similar age range” or “frequently used” should be avoided when selecting atlases. Further, the validity of the networks should be evaluated by computing their associations with behavioral measures.

## 1 Introduction

The process of aging is accompanied by changes in the functional network architecture of the human brain. Overall, there is accumulating evidence for decreased within-network connectivity and increased between-network connectivity in old compared to young adulthood (Deery et al., 2023). However, results have been less consistent in longitudinal studies with older adult samples (Damoiseaux, 2017). Among others, methodological differences between studies hinder generalizable conclusions about aging-related changes (Jockwitz & Caspers, 2021). Given that this field of research is still comparatively young, best practice guidelines or standards have not yet been established. Results may, for example, depend on the spatial definition of the networks and their resolution.

Commonly used methods to estimate resting-state functional connectivity (RSFC) and to extract functional networks encompass seed-based analysis, independent component analysis (ICA), and application of graph theory (Lee et al., 2013;Smitha et al., 2017;van den Heuvel & Hulshoff Pol, 2010). In addition, there are many other machine-learning techniques that can be used to process RSFC data (Khosla et al., 2019). Based on these methods, several large-scale brain networks have been previously described. Some of the reported networks are publicly available in the form of predefined atlases that can be used to calculate the RSFC between voxels or brain regions (also called nodes or parcels). However, these atlases often vary with respect to three major aspects. First, there is variation in the number of predefined networks. For example, atlases with 10 (Smith et al., 2009), 12 (Gordon et al., 2016), 13 (Doucet et al., 2018), or 14 (Shirer et al., 2012) networks were reported. Some atlases even provide networks at different resolutions, for example, seven and 17 networks (Yeo et al., 2011) or 5 and 15 networks (Doucet et al., 2021). Second, the topography of the networks differs. Although the atlases include networks with the same label, the actual network definitions differ with respect to the brain areas involved. For instance,Doucet et al. (2019)compared six different atlases and found that the spatial overlap of networks with homonymous names ranged from 17% to 76%, depending on the network studied, with higher-order networks showing generally low inter-atlas similarity (Doucet et al., 2019). Third, the labeling of the networks is inconsistent. In their meta-analysis,Witt and colleagues (2021)reported nine different labels for networks that appear to be involved in executive functions, with spatially similar networks sometimes being labeled differently and spatially different networks being labeled the same across studies (Witt et al., 2021).

To provide some guidance and harmonization across the different atlases, parcellations, and labels,Uddin and colleagues (2019)proposed a standardization of the taxonomy, including six major networks that can be further divided into subnetworks: the occipital network (ON, “visual network”), the pericentral network (PN, “somatomotor network”), the midcingulo-insular network (M-CIN, “salience network”), the dorsal frontoparietal network (D-FPN, “attention network”), the lateral frontoparietal network (L-FPN, “control network”), and the medial frontoparietal network (M-FPN, “default network”) (Uddin et al., 2019). But even though these networks have been reliably detected in a variety of studies, there is no absolute truth regarding the number of (sub)networks or the definition of the brain areas involved. Consequently, the use of brain atlases has both advantages and disadvantages. On the one hand, they reduce the complexity of the data (e.g., by combining voxels into nodes and/or networks), and, therefore, greatly simplify the comparison of results with previously published findings (Glasser et al., 2016). On the other hand, the atlases may not explain the data very well because they are overfitted (e.g., due to small samples) or because they are based on a population that does not fit the data at hand (Arslan et al., 2018;Khosla et al., 2019;Särelä & Vigário, 2003). The latter is particularly important in the context of aging, considering that most available atlases were created on the basis of young adult samples, typically between the ages of 18 and 35 (Doucet et al., 2019) (seeSupplementary Table 1for an overview).

The application of these atlases to older cohorts implies the generalizability of network solutions to people of older age. However, given that old age is associated with a less segregated network architecture (Deery et al., 2023;Jockwitz & Caspers, 2021), this assumption is not necessarily valid. Indeed,Malagurski et al. (2020b)reported a moderate age effect on promiscuity in the M-FPN, suggesting a tendency for brain regions of the M-FPN to segregate from their “actual” network and connect to other networks with increasing age (Malagurski et al., 2020b). Further,Doucet et al. (2021)reported spatial differences when comparing older versus younger cohorts using an ICA approach. While the posterior medial temporal regions were assigned to the M-FPN in younger adults, these regions were assigned to the L-FPN in older individuals (Doucet et al., 2021).

**Fig. 1.**
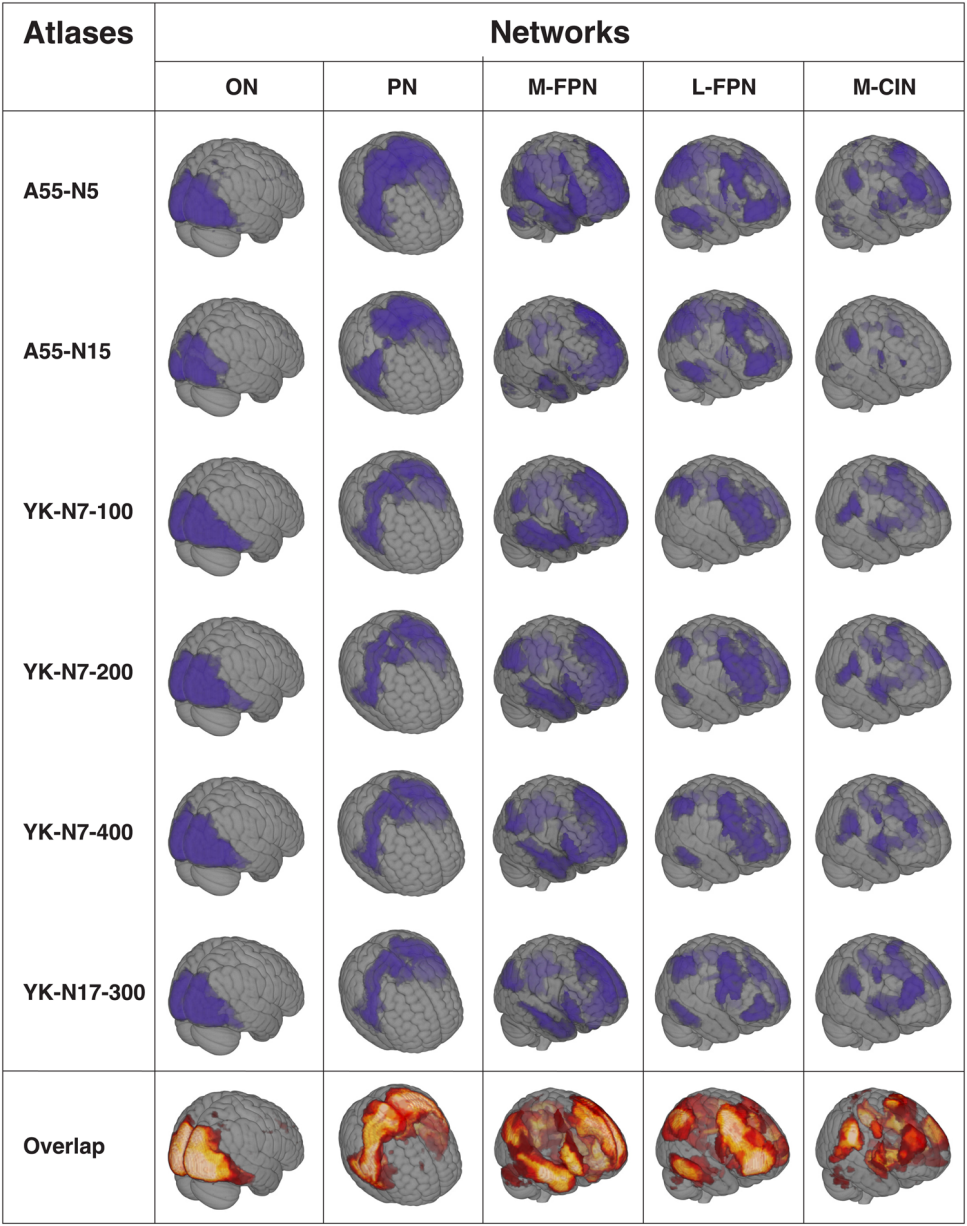
The five binarized brain networks by atlas and overall overlap between the different atlases. The overlap corresponds to the combination of the binarized networks. Light yellow reflects high spatial overlap, whereas darker red represents low spatial overlap. ON = occipital network; PN = pericentral network; M-FPN = medial frontoparietal network; L-FPN = lateral frontoparietal network; M-CIN = midcingulo-insular network; A55 = Atlas55+; YK = Yeo-Krienen Atlas.

From our perspective, to better understand age-related changes in functional brain networks, it is important to assess the impact of differences in spatial network definition on the actual outcomes of interest. Therefore, besides the quantification of spatial overlap between different atlases and their variants, in the current study we primarily explore if and how the differences in network definitions affect the estimated trajectories and, thus, the conclusions about longitudinal changes in RSFC in healthy older adults. To do so, we use two atlases and variants thereof to extract RSFC in five different networks from five measurement occasions covering an observations period of 7 years in a sample of cognitively healthy older adults. On the one hand, we apply the network definitions as implemented in the Yeo-Krienen (YK) atlas (Yeo et al., 2011), which have been the most widely used parcellations in the literature on aging effects on functional network architecture to date (seeSupplementary Fig. 1). On the other hand, we apply the recently published Atlas55+ (A55), which is based on three cohorts of individuals aged 55-95 years and therefore matches the present dataset in terms of age (Doucet et al., 2021).

We expect to replicate smaller network overlaps for higher-order, as compared to primary processing networks. Furthermore, we hypothesize a larger network overlap between different variants of the same atlas compared to the cross-atlas comparisons. With respect to the aging-related changes, we expect that the differences in network definitions will entail differences in the estimated age and time effects, with the scale of differences being a function of spatial overlap of the network definitions. The methodological procedure and the hypotheses were pre-registered before conducting any analyses (https://osf.io/6vrmb). The additional analyses in this study (regional homogeneity, silhouette coefficient, and pooled age and time effects) are labeled as post-hoc analyses.

The results are intended to help estimate the influence of atlas choice on age and time effects and, with this, we hope to inspire the future development of guidelines on the selection of suitable parcellation in the context of network neuroscience and aging.

## 2 Methods

### 2.1 Participants

Data from five measurement occasions (i.e., baseline, 1-year follow-up, 2-year follow-up, 4-year follow-up, 7-year follow-up) were taken from the Longitudinal Healthy Aging Brain Database Project (LHAB; Switzerland) conducted at the University of Zürich (Zöllig et al., 2011). At each measurement occasion, participants underwent brain imaging and completed an extensive battery of neuropsychological, psychometric, and motor tests. Inclusion criteria for study participation at baseline were age ≥ 64, right-handedness, fluent German language proficiency, a score of ≥ 26 on the Mini Mental State Examination (MMSE;Folstein et al., 1975), no self-reported neurological disease of the central nervous system, and no contraindications to magnetic resonance imaging (MRI). The study was approved by the ethical committee of the canton of Zurich, and all participants gave informed consent in accordance with the declaration of Helsinki.

The LHAB dataset includes 232 participants (age at baseline:*M*= 70.85, range = 64–87; females: 114). Self-reported physical and mental health of the sample at baseline, as measured by the SF-12 (Ware et al., 1996), were 50.8 ± 7.4 (*M *± SD) and 54.8 ± 6.2, respectively, which indicates above-average health compared to a normative population (Ware et al., 1995). As expected, these general SF-12 health indicators declined slightly over time, but still indicated above-average health at 7-year follow-up (physical health score: 48.4 ± 8.4, mental health score: 52.9 ± 7.7). At 7-year follow-up, the dataset still comprised 53.88% of the baseline sample (*n*= 125), of which 95% had complete data for resting-state fMRI (*n*= 119). Selectivity analysis revealed that the sample of the 7-year follow-up did not substantially differ from the baseline sample in terms of age, sex, education, or physical and mental health (Supplementary Table 2).

We defined outliers based on the mean framewise displacement (FD) (Power et al., 2012) to increase the quality of the data and to minimize the influence of motion on results. The fMRI data were excluded if the mean FD values at any time point were more than three absolute median deviations (MAD) above the median (Leys et al., 2013). A total of*n*= 40 observations were excluded across all subjects and time points (baseline: 14, 1-year follow-up: 5, 2-year follow-up: 5, 4-year follow-up: 5, 7-year follow-up: 11). This resulted in a sample of 228 participants (age at baseline:*M*= 70.79, range = 64-87; females: 111) with at least one measurement occasion (number of participants: baseline = 209, 1-year follow-up = 201, 2-year follow-up = 185, 4-year follow-up = 157, 7-year follow-up = 108). Of the 228 participants, 90 had five time points, 61 had four time points, 34 had three time points, 22 had two time points, and 21 participants had only one time point.

### 2.2 MRI acquisition

MRI scans were acquired at the University Hospital of Zurich on a Philips Ingenia 3 T scanner (Philips Medical Systems, Best, The Netherlands) using the dsHead 15-channel head coil. T1‐weighted (T1w) structural images were acquired using a gradient echo sequence (3D turbo field echo, 160 slices, TR = 8.18 ms, TE = 3.70 ms, FOV = 240 × 240 × 160 mm, flip angle = 8, isotropic voxel size = 1.0 × 0.938 × 0.938 mm^3^). Two hundred and twenty‐five multislice T2*‐weighted volumes were retrieved within 8 min with a gradient echo‐planar sequence using transverse slice orientation (43 slices; voxel size: 3.5 × 3.44 × 3.44 mm^3^; TR = 2000 ms; TE = 21 ms; flip angle = 76; FOV = 220 × 220 × 150 mm).

### 2.3 MRI preprocessing

Preprocessing was performed with the fmriprep BIDS app (v.1.0.5) (Esteban et al., 2019), a Nipype (Gorgolewski et al., 2011,^2018^) based tool. The T1w volume was corrected for INU (intensity nonuniformity) applying the N4 Bias Field Correction v.2.1.0 (Tustison et al., 2010) and skull-stripped using Advanced Normalization Tools (ANTs) v.2.1.0 (OASIS template). Spatial normalization to the ICBM 152 Nonlinear Asymmetrical template version 2009c (Fonov et al., 2009) was performed by nonlinear registration with the antsRegistration tool of ANTs v.2.1.0 (Avants et al., 2008), using brain-extracted versions of both T1w volume and template. Segmentation of brain tissue into cerebrospinal fluid (CSF), white matter (WM), and gray matter (GM) was performed on brain-extracted T1w using FAST (Zhang et al., 2001) (FSL v5.0.9). Functional data were slice time corrected with 3dTshift from AFNI v.16.2.07 (Cox & Hyde, 1997) and motion corrected with mcflirt (Jenkinson et al., 2002). This was followed by co-registration to the corresponding T1w using boundary-based registration (Greve & Fischl, 2009) with nine degrees of freedom, using flirt (FSL). Motion correcting transformations, BOLD-to-T1w transformation, and T1w-to-template (MNI) warp were concatenated and applied in a single step using antsApplyTransforms (ANTs v.2.1.0) using Lanczos interpolation.

Correlation matrices were estimated with the nilearn Python package (v. 0.7.0) (Abraham et al., 2014). The nuisance regressors were defined according to the 36-parameter model (Ciric et al., 2017): six motion parameters, signals estimated from CSF and WM, global signal, their derivatives, quadratic terms, and squares of derivatives were regressed out from functional data separately for each run. The rs-fMRI data were temporally bandpass filtered in the 0.01–0.1 Hz frequency range. We applied simultaneous filtering/nuisance regression, as it has been reported to reduce the correlation between time-series fluctuations and motion (Hallquist et al., 2013). Global signal regression was performed in accordance with previous studies on aging (Chan et al., 2014;Chong et al., 2019;Malagurski et al., 2020a;Ng et al., 2018), as this has been shown to be effective in the reduction of the effects of physiological signals and head motion (Lydon-Staley et al., 2019).

### 2.4 Network definition and taxonomy

We used the following five networks according to the A55 (Doucet et al., 2021) and the YK (Yeo et al., 2011): ON, PN, M-FPN, L-FPN, and M-CIN. Note that these labels are in line with the taxonomy proposed byUddin et al. (2019), which aims to drive a unified labeling of networks. For information of core regions and equivalent network labels in the atlases, seeTable 1.

**Table 1.** Anatomical network labels and corresponding core regions according toUddin et al. (2019), and labels according to the atlases used.

Anatomical label	Core regions	Label in Doucet et al. (2021)	Label in Yeo et al. (2011)
Occipital Network (ON)	Occipital Lobe, Striate Cortex, Extrastriate Cortex	Visual Network	Visual Network
Pericentral Network (PN)	Motor and Somatomotor Cortices, Anterior and Posterior Central Sulcus, Juxtapositional Lobule	Sensorimotor Network	Somatomotor Network
Medial Frontoparietal Network (M-PFN)	Medial Prefrontal Cortex, Posterior Cingulate Cortex, Inferior Parietal Lobule, Inferior Frontal Gyrus, Middle Temporal Gyrus, Superior Temporal Sulcus, Parahippocampal Cortex	Default Mode Network	Default Mode Network
Lateral Frontoparietal Network (L-PFN)	Lateral Prefrontal Cortex, Middle Frontal Gyrus, Anterior Inferior Parietal Lobe, Midcingulate Gyrus	Executive Control Network	Frontoparietal Network
Midcingulo-insular Network (M-CIN)	Bilateral Anterior Insula, Anterior Midcingulate Cortex	Salience Network	Ventral Attention Network

**Fig. 2.**
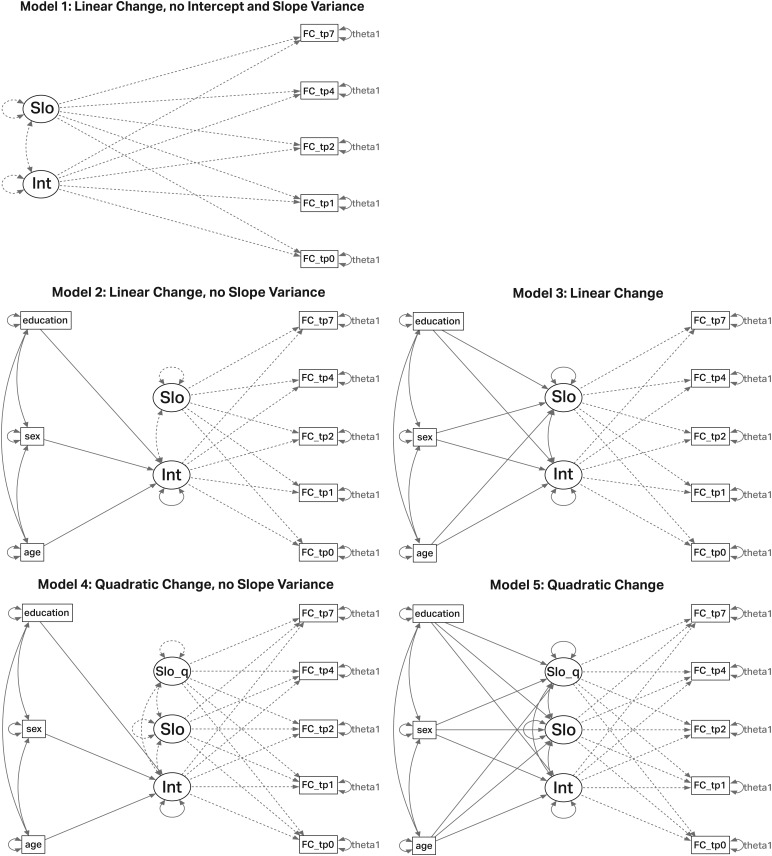
Visualization of the five fitted models. Latent factors are shown as a circle and manifest variables as a square. Factor loadings for latent intercept were set to 1 and for latent slope to 0,1,2,4,7 (according to elapsed time since study start). Dashed lines for the latent variables mean that variance and the covariance were set to zero (models in the left column). Theta 1 refers to the error variance and was kept constant for each time point. FC = functional connectivity; Int = Intercept; Slo = linear Slope; Slo_q = quadratic Slope; FC = resting-state functional connectivity; tp = time point.

**Fig. 3.**
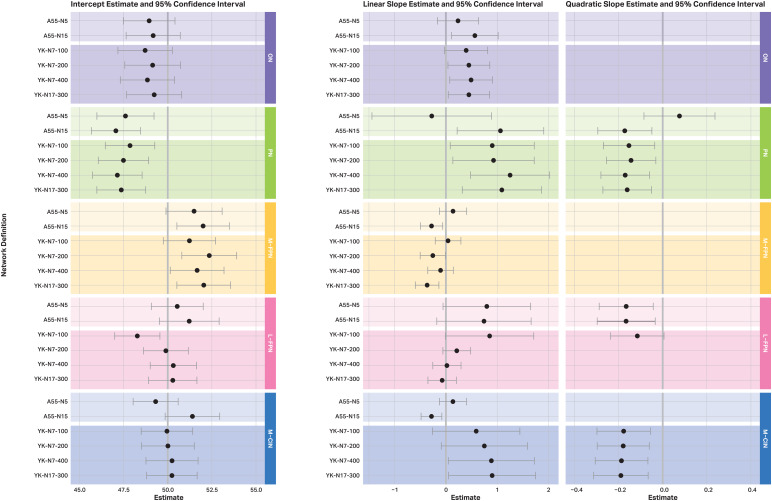
Estimated intercept, linear and quadratic slope, and their 95% confidence intervals for each network by atlas. The unit of the values correspond to T-scores (*M*= 50, SD = 10). The grey line at 50 (on the left - intercept estimate) marks the mean value of the T-score. The two grey lines at 0 (on the right - slope estimates) mark the limit that the confidence intervals must exceed for the linear and/or quadratic slope estimate to be significant at the 5% alpha level. For example, the linear slope in the ON is not significant using the A55-N5, whereas a significant linear increase is estimated using the A55-N15 (grey line at 0 is not included in the interval). If the confidence intervals of the estimates do not overlap between the atlases, this indicates a significant difference in the estimate at the 5% alpha level. For example, when comparing the confidence intervals of the linear slopes in the ON, it is evident that all intervals overlap. Hence, there seem to be no systematic differences in the linear slope estimates depending on the atlas used. Note that depending on which model showed the best fit, certain effects and their confidence intervals are not present (e.g., if the linear model showed the best fit for a given network and atlas, no effects are shown for the quadratic change, as this was not estimated). ON = occipital network; PN = pericentral network; M-FPN = medial frontoparietal network; L-FPN = lateral frontoparietal network; M-CIN = midcingulo-insular network; A55 = Atlas55+; YK = Yeo-Krienen Atlas, N = network.

We chose the A55 because it was generated based on three cohorts of older adults and consequently matches the sample used in the present analysis in terms of age. Furthermore, we chose the YK for comparison because it is the most used atlas in the field of network neuroscience in healthy aging.Supplementary Text 1andSupplementary Figures 1-3provide an overview of a literature review we conducted on the topic of resting-state networks and healthy aging to illustrate the range of methodologies used.

**Fig. 4.**
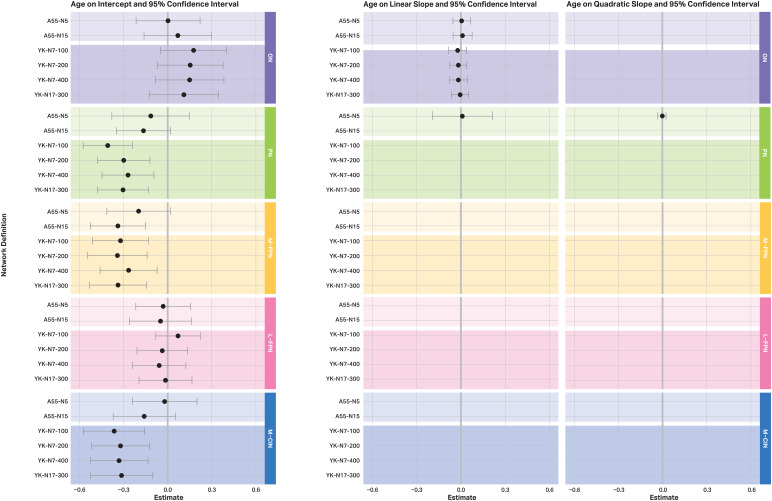
Estimated age effect on intercept, linear and quadratic slope, and their 95% confidence intervals for each network by atlas. The unit of the values corresponds to T-scores (*M*= 50, SD = 10). The grey lines at 0 mark the limit that the confidence intervals must exceed for an age effect to be significant at the 5% alpha level. For example, the age effect on the intercept in the M-FPN is not significant using the A55-N5 (grey line is included in the interval), whereas a significant negative age effect is estimated using the A55-N15 (grey line at 0 is not included in the interval). That is, older individuals show lower baseline FC in the M-FPN using the A55-N15, but not when using the A55-N5. If the confidence intervals of the estimates do not overlap between the atlases, this indicates a significant difference in the estimate at the 5% alpha level. For example, when comparing the confidence intervals of the age effects on the intercept in the M-FPN, it is evident that all intervals overlap. Hence, there seem to be no systematic differences in the estimated age effects depending on the atlas used. Note that depending on which model showed the best fit, certain effects and their confidence intervals are not present (e.g., if the linear model showed the best fit for a given network and atlas, no effects are shown for the quadratic change, as this was not estimated). ON = occipital network; PN = pericentral network; M-FPN = medial frontoparietal network; L-FPN = lateral frontoparietal network; M-CIN = midcingulo-insular network; A55 = Atlas55+; YK = Yeo-Krienen Atlas, N = network.

For A55, we used both available atlas variants: the five-network variant (A55-N5) comprising ON, PN, M-FPN, L-FPN, and M-CIN and the 15-network variant (A55-N15), in which the five networks are divided into subnetworks (seeTable 2andSupplementary Fig. 4). Note that we are referring to variants rather than resolution because with the A55-N15 we are not looking at the individual subnetworks. Instead, the subnetworks are combined to the five main networks. To calculate within-network RSFC, we used the division into nodes according to the automated anatomical labeling 3 atlas (AAL3) (Rolls et al., 2020), which consists of 166 nodes in total. We therefore multiplied the binary masks of the networks by the AAL3 atlas to obtain distinct nodes. This resulted in a total number of 411 nodes for the A55-N5 and 294 for the A55-N15.

**Table 2.** Number and labels of subnetworks for Atlas55+ with 15 networks (A55-N15) and Yeo-Krienen atlas with 17 networks (YK-N17-300).

	Atlas	
Network	A55-N15	YK-N17-300
Occipital Network (ON)	Subnetwork 1: Medial Visual Subnetwork 2: Posterior Visual	Subnetwork 1: Visual Central Subnetwork 2: Visual Peripheral
Pericentral Network (PN)	Subnetwork 1: Ventral SMN Subnetwork 2: Supplementary Motor Area Subnetwork 3: Left SMN Subnetwork 4: Auditory	Subnetwork 1: Somatomotor A Subnetwork 2: Somatomotor B
Medial Frontoparietal Network (M-PFN)	Subnetwork 1: Main DMN Subnetwork 2: Anterior DMN Subnetwork 3: Language 1 Subnetwork 4: Language 2	Subnetwork 1: Default A Subnetwork 2: Default B Subnetwork 3: Default C
Lateral Frontoparietal Network (L-PFN)	Subnetwork 1: Right ECN Subnetwork 2: Left ECN Subnetwork 3: Medial Temporal Lobe Subnetwork 4: Dorsal Attention	Subnetwork 1: Control A Subnetwork 2: Control B Subnetwork 3: Control C
Midcingulo-insular Network (M-CIN)	Subnetwork 1: Salience	Subnetwork 1: Ventral Attention A Subnetwork 2: Ventral Attention B

An exemplary visualization of the occipital subnetworks can be found inSupplementary Figure 4.

Similarly, we used both the seven (YK-N7) and 17 network (YK-N17) variants of the YK atlas. Besides ON, PN, M-FPN, L-FPN, and M-CIN, the 7-network variant additionally contains a dorsal attention and a limbic network. The latter, however, were not considered in the current analyses. As for the A55-N15, networks are split-up into subnetworks in the 17-network variant (seeTable 2). Here again, the subdivisions for each network have been combined into one network. To calculate the within-network RSFC, we applied the available divisions into 400 nodes for the seven-network-solution (YK-N7-400) and the division into 300 nodes for the 17-network-solution (YK-N17-300) according to the Schaefer atlas (Schaefer et al., 2018). The number of nodes was chosen to be equal to the total number of nodes used in the A55. However, to additionally assess the impact of the number of nodes, we also computed within-network RSFC using the 100 (YK-N7-100) and 200 (YK-N7-200) nodes for the five-network resolution of the YK. A visualization of the networks and their overall overlap can be found inFigure 1*.Supplementary Figure 4shows an exemplary visualization of the occipital subnetworks of the A55-N15 and YK-N17 and their nodes.*Mean within-network RSFC was estimated by averaging the correlations between all nodes belonging to the network of interest and transforming them into*T*-scores (*M*= 50, SD = 10 at baseline). This resulted in a total of six datasets (two for A55 and four for YK) for each of the five networks.

### 2.5 Statistical analysis

The spatial overlap of the networks was quantified based on the dice similarity coefficient (DSC) (Dice, 1945). The DSC is defined as twice the union of the voxels (voxels selected as belonging to the network in both atlases) divided by the sum of the voxels from the two atlases marked as belonging to the network. It provides information about the extent to which the networks are spatially similar, with 0 indicating no overlap and 1 indicating perfect overlap. We calculated a Kruskall-Wallis test (Kruskal & Wallis, 1952) to make comparisons between inter- and intra-atlas DSC, and between the DSC of higher-order and primary processing networks. We also applied post-hoc Wilcoxon-Mann-Whitney tests (Mann & Whitney, 1947;Wilcoxon, 1947) with Bonferroni correction to compare the networks individually.

The longitudinal data were analyzed with latent growth curve (LGC) models in R version 4.1.0 (R Core Team, 2020) using the lavaan package version 0.6-12 (Rosseel, 2012). For each of the 30 datasets (5 networks * 6 operationalizations), we computed five models of varying complexity (for Visualization, seeFig. 2). Our aim was to describe the data over time as simple as possible, but as complex as necessary. We therefore varied two factors that are relevant for longitudinal modeling: (1) Is the change over time linear or nonlinear? (2) Is there variability between individuals (in the change over time), or is the main effect sufficient to describe the data? The first model was the simplest, consisting of an intercept and a linear slope, with between-person variance and covariance between intercept and slope set to zero (main effects only). The second model was an extension of the first, with between-person variance estimated only for the intercept (random effect). In addition, regressions of age at entry, sex, and education on the intercept were calculated to explain part of the potential variance in the intercept. In the third model, between-person variance was calculated for both the intercept and the linear slope (random effects), allowing the covariance between intercept and linear slope to be estimated as well. Again, the regressions of age at entry, sex, and education on intercept and slope were calculated. The fourth model consisted of an intercept and two slopes (linear and quadratic), with between-person variance only allowed in the intercept, including the above-mentioned regressions on the intercept. The fifth model was the most complex, consisting of an intercept, a linear slope, and a quadratic slope. For all three factors, the between-person variances and the factor covariances were estimated, and regressions of the three covariates on the factors were included. The factor loadings for the latent variables were defined as follows: For the intercept, they were fixed at one (1,1,1,1,1); for the linear slope, they were fixed according to the time intervals (0,1,2,4,7); and for the quadratic slope, the factor loadings of the linear slopes were squared (0,1,4,16,49). The three time-invariant covariates were coded as follows: age at study entry (mean centered, 0 = 70.84 years), sex (0 = female, 1 = male), and education (0 = medium level). The residual means were fixed to zero, and the residual variance (theta) was held constant over time.

The evaluation of correspondence between different atlases followed a two-step approach. In the first step, we compared whether the same model best described the data. To find the best model, six goodness-of-fit criteria were considered in each case. We used the ratio of the$\chi^{2}$-test to the respective degrees of freedom ($\chi^{2}$/df) (Jöreskog & Sörbom, 1993;Marsh & Hocevar, 1985), the*p*-value of the Chi-Statistic, the Comparative Fit Index (CFI) (Bentler, 1990), the root mean square error of approximation (RMSEA) (Browne & Cudeck, 1992;Steiger & Lind, 1984), the Bayesian Information Criterion (BIC) (Neath & Cavanaugh, 2012), and the occurrence of a Heywood case (Kolenikov & Bollen, 2012).

The fit criteria were considered good when:$\chi^{2}$/ df ≤ 2,*p*-value > 0.05, CFI > 0.97, RMSEA ≤ 0.05, and acceptable fit was defined as:$\chi^{2}$/df ≤ 3,*p*-value > 0.01, CFI > 0.95, RMSEA ≤ 0.08 (Hu & Bentler, 1998;Jöreskog & Sörbom, 1993;Schermelleh-Engel et al., 2003). For each data set, the model that had the most indicators classified as good was chosen. If there were several models eligible, the one with the smallest BIC was chosen, as BIC penalizes model complexity and therefore reduces overfitting (Vrieze, 2012). Furthermore, model solutions with Heywood cases were considered as bad fit, as negative variances are not possible by definition and may be an indicator of model misspecification (Kolenikov & Bollen, 2012).

In the second step, we compared the 95% confidence intervals of the estimated parameters to see if estimates of intercepts, slopes, their potential correlations, and regressions overlapped between different atlases. The potential non-overlap may indicate that the estimation of a parameter differs systematically depending on the atlas used. Note that the procedure implies a*p*-value of 0.05 and that no correction for multiple testing has been applied. So far, there is little consensus on how to correct*p*-values in complex structural equation models, which is why many researchers do not apply any correction (Cribbie, 2007;C. E. Smith & Cribbie, 2013). The proposed adjusted Bonferroni correction bySmith and Cribbie (2013), which takes into account the covariance structure of the data, would lead to different thresholds of the*p*-values for each model, so that differences would no longer be attributable to the atlas choice alone, but also to the correction procedure of the*p*-values. Therefore, we decided to leave the*p*-value at 5%.

Because the selectivity analysis did not indicate a systematic drop-out, we assumed missing values to be missing at random (MAR) (Little, 1995) and applied Full Information Maximum Likelihood Estimation (FIML) (Finkbeiner, 1979;Schafer & Graham, 2002) to preserve as much data as possible.

### 2.6 Post-hoc analysis of atlas fit

To evaluate which atlas better suits our data, we additionally calculated the widely used regional homogeneity (ReHo) coefficient (Craddock et al., 2012;Gordon et al., 2016;Zang et al., 2004) and the silhouette coefficient (Rousseeuw, 1987;Yeo et al., 2011). For ReHo, the assumption is that a good parcellation is able to combine those voxels with similar BOLD signal over time into nodes, thus meaning that the BOLD signal in a voxel should behave similarly to the BOLD signal of the neighboring voxels. We calculated the Kendall’s coefficient of concordance (KCC) (Kendall, 1957;Kendall & Smith, 1939) for the BOLD-signal fluctuations of each voxel with its 26 neighboring voxels using AFNI’s 3dReHo (Taylor & Saad, 2013) command. The formula is defined as follows:

$$ReHo=W=\frac{\sum {\left(R_{i}\right)}^{2}-n{\left(\bar{R}\right)}^{2}}{\frac{1}{12}K^{2}\left(n^{3}-n\right)}$$

where*W*is the KCC of an individual voxel with a range from 0 to 1, with 0 indicating no similarity and 1 indicating perfect coherence;*R_i_*is the sum rank of the*i*^th^time point;$\bar{R}$= (*n*+1)*K*/2 is the mean of the*R_i_*;*K*is the number of time series within a measured cluster (*K*) (here:*K*= 27, the given voxel plus the 26 neighbors); and*n*is the number of ranks (here: 225, as 225 volumes were used).

Similarly, the silhouette coefficient (SICO) is a measure of how well a parcellation fits the data. In contrast to ReHo, however, it is not a quality measure for the local averaging of the BOLD signals (i.e., the definition of nodes), but concerns the allocation of the nodes to the networks (or clusters). We calculated the SICO as follows:

$$SICO_{i}=\frac{b_{i}-a_{i}}{\text{max}\left(a_{i},b_{i}\right)}$$

where*a_i_*represents the average dissimilarity of the*i*^th^node to the nodes assigned to the same network and*b_i_*represents the average dissimilarity of the*i*^th^node to the nodes assigned to the closest of the four other networks (i.e., to the network with the lowest dissimilarity).*a_i_*and b*_i_*are defined as follows:

$$a_{i}=\frac{1}{n_{k}-1}\sum_{j\in U_{k},i\neq j}1-r(v_{i},v_{j}),b_{i}=\frac{1}{M}\sum_{j\in \mathbb{N}(U_{k})}1-r(v_{i},v_{j})$$

where*r*(*v*_i_,*v*_j_) is the functional connectivity between node*i*and node*j*, with 1 —*r*(*v*_i_,*v*_j_) representing the dissimilarity between node*i*and node*j, U_k_*is the set of nodes in network*k*, and*v*_i_is an element of the set*U_k_*.*n_k_*is the number of nodes in the network*k*.ℕ(*U_k_*) is the set of nodes in the closest network of network*k*, and*M*is the total number of nodes in this network. The silhouette coefficient ranges from -1 to 1, where -1 indicates that the node should be assigned to the other network, 0 means that the node fits both networks (ambiguous), and 1 means that the node is well assigned.

Null models to determine the ReHo and SICO under chance were created using the spin-test (Alexander-Bloch et al., 2018). We therefore projected the volumetric atlases and the baseline data (rs-fMRI and ReHo maps) onto the Freesurfer (Fischl, 2012) average surface with 10242 vertices per hemisphere (i.e., 20482 vertices total). For this we used the command*mni152_to_fsaverage*(Buckner et al., 2011;Wu et al., 2018) from the*neuromaps*toolbox (Markello et al., 2022) in Python. For the atlases, nearest neighbor interpolation was used, whereas linear interpolation was used for the MRI data. Next, we used the*nulls.alexander_bloch*(Alexander-Bloch et al., 2018) command to compute the rotated atlases. In short, the atlas projected to the surface is transformed to a sphere and then randomly rotated (in our case 100 times) so that an atlas with similar properties (shape and location of the nodes) is generated. The ReHo and SICO values using the actual atlases were then compared to the values given by the randomly rotated atlases to determine if the parcellation is significantly better than a random parcellation with similar spatial features as the original atlas. Note that the subcortical and cerebellar structures of A55-N5 and A55-N15 were omitted. For both, the ReHo and SICO, we averaged the values of the individual nodes for each of the five networks. We then calculated pairwise comparisons on the baseline sample using the Wilcoxon-Mann-Whitney test to estimate potential differences in the coefficients between the atlases and their variants. We further correlated the coefficients with age to test whether an atlas/variant tends to have higher values for older individuals. The*p*-values for comparisons and correlations were adjusted using the Bonferroni-correction.

**Fig. 5.**
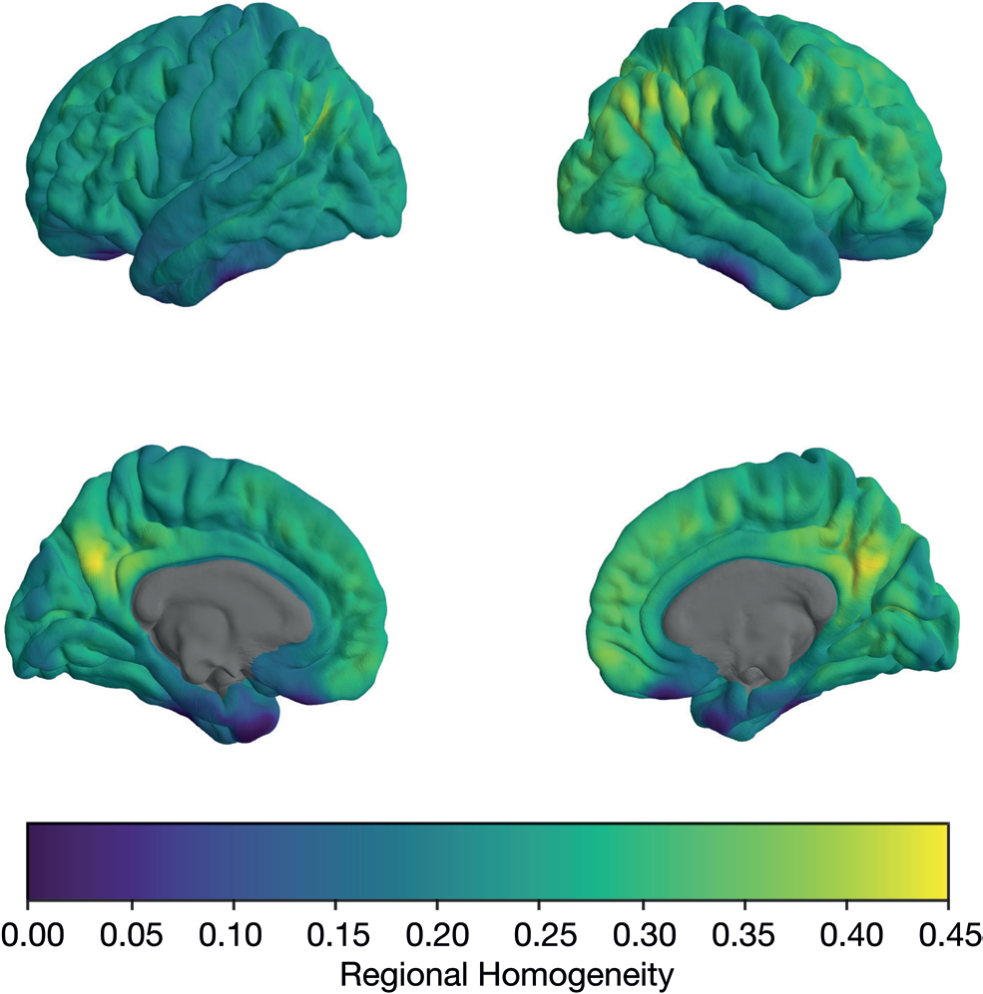
Mean regional homogeneity map of baseline data (*N*= 209) projected on Freesurfer average surface. The figure shows the averaged regional homogeneity maps of the baseline sample (*N*= 209). The mean of the overall ReHo values across the cortex is 0.251 with a standard deviation of 0.040. ReHo values can range from 0 to 1, where 0 means that on average there is no coherence of the BOLD signals between the voxels or vertices and 1 means perfect coherence. The figure shows clear variability across the cortex. Particularly high values are observed in the medial frontal cortex, posterior cingulate cortex, precuneus, and parietal areas. Lower values can be found in the (left) motor and sensory cortex, orbito-frontal cortex, as well as in temporal regions.

Note that for the A55-N5 and A55-N15, we additionally used the nodes by Schaefer (i.e., the nodes we used for the YK). This is because both ReHo and SICO depend on the node definition. As the Schaefer’s nodes are based on functional data, they tend to have a higher ReHo, which may also affect the SICO. For this purpose, we assigned Schaefer’s 400 nodes to the A55-N5 networks and Schaefer’s 300 nodes to the A55-N15 networks (to match the number of nodes with the number of AAL3 nodes we used). We achieved this by calculating the DSC for each node to the five networks, and counting the node to the network where the DSC was highest. Finally, we calculated the DSC between the A55 networks as defined with the Schaefer nodes and the original A55 networks to see how much the spatial definition changes (seeSupplementary Fig. 5). This spatial change is due to the fact that the Schaefer nodes do not cover subcortical and cerebellar parts. Furthermore, the nodes do not follow the network boundaries of the A55, so that the network boundaries shift to some extent.

### 2.7 Post-hoc analysis of pooled effects

Since the same trajectory was estimated for three networks across all atlases (e.g., linear change over time), we did post-hoc analyses to estimate a pooled effect of time for ON, PN, and M-FPN. Additionally, we estimated the pooled effects of age on the intercept and the slope (only for the ON, as it was the only network showing variance in the slope). We therefore weighted the estimate of the effects by the inverse of the squared variance of the effect (Chang et al., 2022). This method results in a weighting of the estimates according to their certainty, that is, the higher the standard error of the estimate, the lower the weighting for the pooled effect.

## 3 Results

### 3.1 Spatial overlap between network definitions

**Fig. 6.**
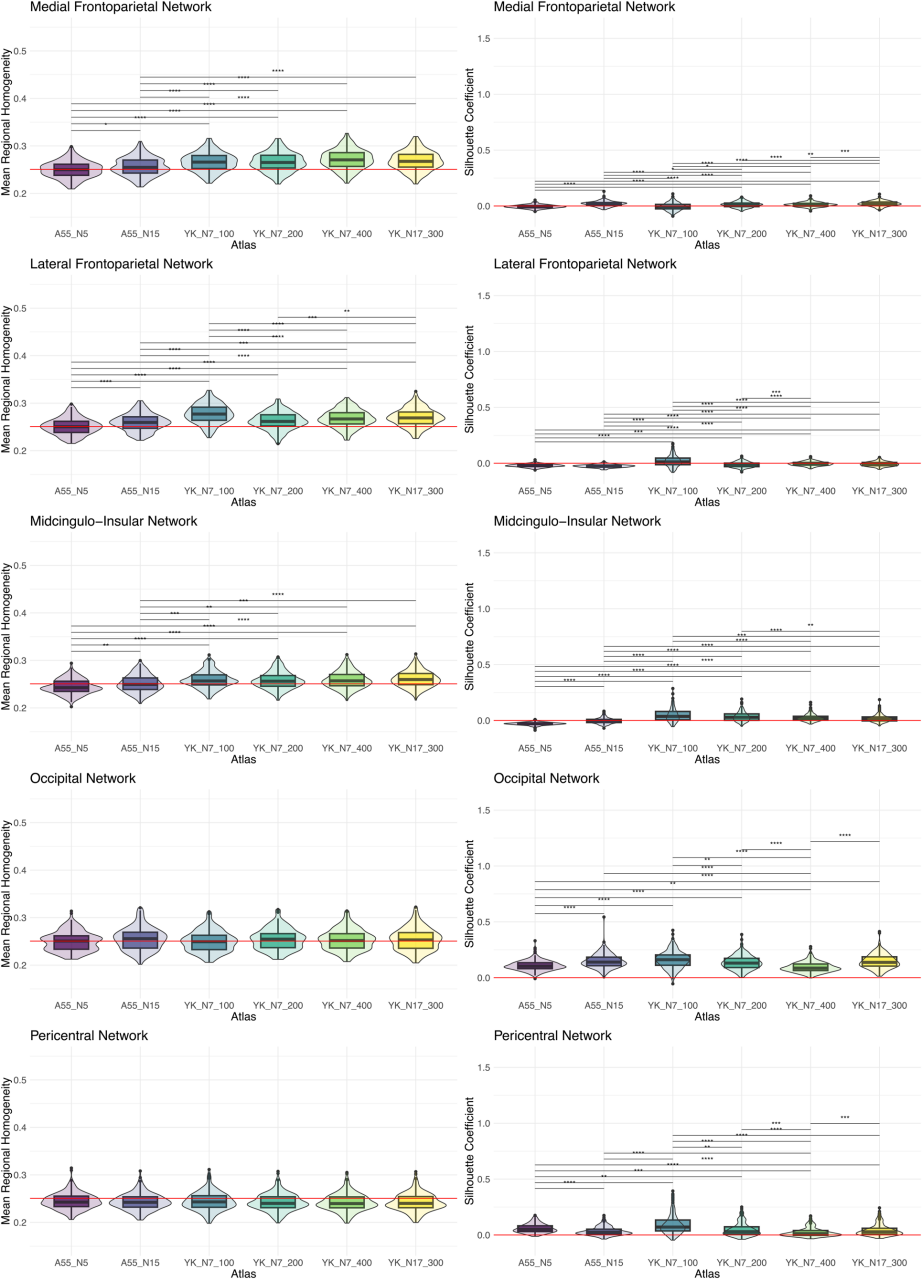
Regional homogeneity and silhouette coefficient by network and atlas with Bonferroni corrected results of pairwise comparisons between atlases using Wilcoxon-Mann-Whitney test. The average regional homogeneity can range from 0 to 1, where 0 means that on average there is no coherence of the BOLD signals between the voxels assigned to the network and 1 means perfect coherence. The silhouette coefficient can range from -1 to 1, where -1 means that the nodes should be better assigned to another network, 0 means that on average the nodes fit two networks equally well (ambiguous), and 1 means that on average the nodes fit the assigned network very well. The red lines in the left plots (ReHo) indicate the average ReHo value across the cortex (*M*= 0.251). The red lines in the right plots (SICO) are placed at 0. Values above the line indicate an acceptable allocation of the nodes to the networks. A55 = Atlas55+; YK = Yeo-Krienen Atlas.

Spatial overlap metrics (i.e., DSC) are summarized inTable 3andSupplementary Figure 6. Median DSC across all comparisons was 0.67, ranging from 0.20 to 0.93. The Kruskal-Wallis test revealed significant differences (χ^2^(4) = 8.520,*p*< .001) between the networks. Pairwise post-hoc Wilcoxon rank-sum tests with Bonferroni correction (α = 0.05/10 or*p*-value*10) indicated significant differences in DSC between L-FPN and PN (*W*= 182,*p*_adj_= .032), L-FPN and ON (*W*= 198,*p*_adj_= .002), and M-CIN and ON (*W*= 191,*p*_adj_= .007). Furthermore, there were significant differences in the inter- and intra-atlas comparisons (*W*= 125,*p*< .001) and between primary processing and higher-order networks (*W*= 259,*p*< .001) when grouped together.

**Table 3.** Median dice similarity coefficient by network and in total.

	Networks					Total
	Primary processing		Higher-order			
	ON	PN	M-FPN	L-FPN	M-CIN	
Median	0.77 ^a,b^	0.70 ^c^	0.60	0.50 ^a,c^	0.41 ^b^	0.67
Range	0.63 – 0.93	0.60 – 0.91	0.53 – 0.83	0.34 – 0.78	0.20 – 0.80	0.20 – 0.93
Intra Atlas	0.92	0.87	0.81	0.72	0.72	0.80 ^d^
Inter Atlas	0.72	0.66	0.58	0.42	0.30	0.58 ^d^

Comparisons based on Kruskall-Wallis test and post-hoc test using Wilcoxon-Mann-Whitney-Test with Bonferroni correction. Significant differences in dice similarity coefficient between:^a^ON and L-FPN (*W*= 198,*p*_adj_= .002);^b^ON and M-CIN (*W*= 191,*p*_adj_= .007);^c^PN and L-FPN (*W *= 182,*p*_adj_= .032);^d^Intra- and Inter Atlas comparisons (*W*= 125,*p*< .001).

ON = occipital network; PN = pericentral network; M-FPN = medial frontoparietal network; L-FPN = lateral frontoparietal network; M-CIN = midcingulo-insular network.

### 3.2 Effect of network definition on model selection

Results of model selection are shown inTable 4. Detailed results, including model fit indices, occurrence of Heywood cases, and model estimated including*p*-values can be found inSupplementary Tables 3 and 4. The model selection process revealed no differences in terms of the trajectory for the ON (linear), PN (quadratic), and M-FPN (linear). In contrast, the trajectories for L-FPN and M-CIN varied depending on the atlas used. When RSFC strength is extracted based on the A55 atlas, a quadratic change is assumed for the L-FPN and a linear change for the M-CIN, while it is the other way round when using the YK. Further, there was a difference for slope variance in the PN. Using the A55-N5, the quadratic model with slope variance showed the best fit, while the quadratic model without slope variance revealed better fit for other atlas variants.

**Table 4.** Model selection for each atlas and network.

Atlas	ON		PN		M-FPN		L-FPN		M-CIN	
	Slope	Slope variance	Slope	Slope variance	Slope	Slope variance	Slope	Slope variance	Slope	Slope variance
A55-N5	Linear	Yes	Nonlinear	Yes	Linear	No	Nonlinear	No	Linear	No
A55-N15	Linear	Yes	Nonlinear	No	Linear	No	Nonlinear	No	Linear	No
YK-N7-100	Linear	Yes	Nonlinear	No	Linear	No	Nonlinear	No	Nonlinear	No
YK-N7-200	Linear	Yes	Nonlinear	No	Linear	No	Linear	No	Nonlinear	No
YK-N7-400	Linear	Yes	Nonlinear	No	Linear	No	Linear	No	Nonlinear	No
YK-N17-300	Linear	Yes	Nonlinear	No	Linear	No	Linear	No	Nonlinear	No

ON = occipital network; PN = pericentral network; M-FPN = medial frontoparietal network; L-FPN = lateral frontoparietal network; M-CIN = midcingulo-insular network; A55 = Atlas55+; YK = Yeo-Krienen Atlas, N = network.

### 3.3 Comparison of confidence intervals for trajectories and age effects

Estimates and 95% confidence intervals of intercepts, linear slopes, and quadratic slopes are displayed inFigure 3. Estimates of age effects on intercepts, linear slopes, and quadratic slopes are displayed inFigure 4. Effects of sex and education can be found inSupplementary Figures 7 and 8. Generally, the confidence intervals for the intercepts and the slopes, as well as for the age effects, overlapped except for the linear slope in the M-FPN. There is a non-significant increase in RSFC over time when using the A55-N5 (95% CI: -0.127; 0.405), whereas there is a significant decrease when using the YK-N17-300 (95% CI: -0.596; -0.136).

### 3.4 Post-hoc analysis of atlas fit

A visualization of the averaged ReHo map of the baseline data is shown inFigure 5. The mean ReHo value is*M*= 0.251 (SD = 0.040), with variability across the entire cortex, showing ReHo values up to 0.450 (especially in areas of the M-FPN). The*p*-values of ReHo and SICO values for each atlas based on the spin-test are summarized inTable 5. The distributions and pairwise comparisons using Wilcoxon-Mann-Whitney test for ReHo and SICO are shown inFigure 6. Median and range for each network and correlations with age are summarized inSupplementary Tables 5 and 6.

**Table 5.** Summary of*p*-values obtained by spin test for regional homogeneity and silhouette coefficient by network.

Regional homogeneity						
Atlas	Mean	ON	PN	M-FPN	L-FPN	M-CIN
A55-N5	*p* = .80	*p* = .65	*p* = .67	*p* = .61	*p* = .55	*p* = .78
A55-N15	*p* = .31	*p* = .58	*p* = .64	*p* = .35	***p*** ** = .05**	*p* = .51
YK-N7-100	***p*** ** = .00**	*p* = .67	*p* = .65	***p*** ** = .05**	***p*** ** = .01**	*p* = .23
YK-N7-200	***p*** ** = .03**	*p* = .61	*p* = .64	***p*** ** = .02**	*p* = .06	*p* = .20
YK-N7-400	***p*** ** = .01**	*p* = .62	*p* = .63	***p*** ** = .00**	***p*** ** = .00**	*p* = .21
YK-N17-300	***p*** ** = .00**	*p* = .60	*p* = .64	***p*** ** = .00**	***p*** ** = .00**	*p* = .19

Silhouette coefficient						
Atlas	Mean	ON	PN	M-FPN	L-FPN	M-CIN
A55-N5	***p*** ** = .01**	***p*** ** = .05**	*p* = .17	***p*** ** = .00**	***p*** ** = .00**	*p* = .14
A55-N15	***p*** ** = .02**	*p* = .11	*p* = .13	***p*** ** = .00**	***p*** ** = .00**	*p* = .08
YK-N7-100	***p*** ** = .00**	*p* = .09	*p* = .16	***p*** ** = .01**	***p*** ** = .00**	***p*** ** = .00**
YK-N7-200	***p*** ** = .00**	*p* = .06	*p* = .13	***p*** ** = .00**	***p*** ** = .02**	***p*** ** = .04**
YK-N7-400	***p*** ** = .00**	*p* = .10	*p* = .12	***p*** ** = .00**	***p*** ** = .03**	***p*** ** = .00**
YK-N17-300	***p*** ** = .00**	*p* = .06	*p* = .11	***p*** ** = .00**	***p*** ** = .00**	*p* = .07

The mean refers to the averaged ReHo and SICO values across the networks, whereby the averaged empirical values were then compared with the averaged null model values.

The results of the spin test show that the four variants of the YK atlas have significantly higher ReHo values compared to the rotated atlases when averaged across all five networks, whereas the two variants of the A55 do not. At the network level, it is evident that the nodes of the M-FPN and L-FPN in particular are well defined, as these networks have significantly higher ReHo values than when applying the rotated atlases. This is also true for the L-FPN using the A55-N15. However, it should be noted that the interpretation at network level should be treated with caution, as there is variability of ReHo across the cortex. Hence, networks that tend to have below-average ReHo values (e.g., PN and ON, seeFigs. 5and6) have only a small chance of becoming significant, as the random rotation of the atlases increases the probability that brain regions with above-average ReHo values will be included for the null models (e.g., brain regions such as the medial frontal cortex or the precuneus). The reverse is also true, that is, the probability of networks with high empirical ReHo becoming significant is greater, since rotation increases the probability that brain areas with low ReHo are included in the null models. However, the results of the Wilcoxon-Mann-Whitney test show that the ReHo values for the three higher-order networks are significantly higher when using the YK variants compared to the A55 variants. Thus, there are differences in the fit of the atlases to the data, with the higher-order networks showing better fit for the YK variants than for the A55 variants, whereas there are no differences for the primary processing networks.

For SICO, a significant value is obtained for all atlases when averaged across the five networks. That is, for all atlases, the allocation of the nodes to the networks is significantly better than a random allocation. At the network level, it is evident that the nodes of the higher-order networks were allocated particularly well compared to the randomly rotated atlases. The exact median values of the SICO show that the higher-order networks in particular fluctuate around 0 for all atlases, whereas the primary processing networks tend to show higher SICO medians and also a larger range (seeSupplementary Table 5). The Wilcoxon-Mann-Whitney test revealed that highest SICO values for the M-FPN are achieved using the A55-N15 or YK-N17-300, as these two showed significantly higher values than the other atlases. For the other networks, the highest values were achieved using the YK-N7-100, despite not always being significantly higher than the second-best atlas (e.g., ON). Using the Schaefer nodes for the two A55 variants slightly improved the ReHo and SICO values, but overall the YK variants still showed higher values than the A55.

The associations of ReHo and SICO with age revealed only one significant correlation between age and the ReHo in the M-FPN of the A55-N5 (*r_s_*= 0.184,*p_adj_*= 0.046) after Bonferroni correction. The additional analyses for the A55 with nodes by Schaefer can be found inSupplementary Figure 9andSupplementary Tables 7 and 8.

### 3.5 Post-hoc analysis of pooled effects

The post-hoc pooled effects for age and time are summarized inTable 6. Significant changes in connectivity over the 7 years were found in ON and PN, while the connectivity within the M-FPN remained stable. Further, significant age effects on the intercept were found for the PN, M-FPN, and M-CIN, while for the ON and L-FPN, the effects were not statistically significant.

**Table 6.** Pooled estimates and 95% confidence intervals for time and age effects.

Network	Linear slope		Quadratic slope		Age on intercept		Age on linear slope	
	Estimate	95% CI	Estimate	95% CI	Estimate	95% CI	Estimate	95% CI
ON	**0.425**	**0.010; 0.841**	-	-	0.109	-0.118; 0.336	-0.008	-0.068; 0.052
PN	**0.938**	**0.108; 1.768**	**-0.138**	**-0.256; -0.020**	**-0.280**	**-0.462; -0.097**	-	-
M-FPN	-0.157	-0.398; 0.085	-	-	**-0.304**	**-0.501; -0.108**	-	-
L-FPN	-	-	-	-	-0.012	-0.189; 0.165	-	-
M-CIN	-	-	-	-	**-0.259**	**-0.465; -0.052**	-	-

The pooled effects were calculated by weighting the estimates by the inverse of the squared variance of the estimate. The values in bold indicate significance. Dashes indicate that the pooled effects could not be estimated (e.g., age effects on the linear slope were only possible for ON, as there was no variance in the slope for the remaining networks).

ON = occipital network; PN = pericentral network; M-FPN = medial frontoparietal network; L-FPN = lateral frontoparietal network; M-CIN = midcingulo-insular network; A55 = Atlas55+; YK = Yeo-Krienen Atlas, N = network, CI = confidence interval.

## 4 Discussion

The current study investigated the congruence of different spatial definitions of five functional brain networks, which were adopted from the Yeo-Krienen atlas and the Atlas55+ (Doucet et al., 2021;Yeo et al., 2011). Secondly, using a longitudinal dataset, the study explored if the association between age and RSFC and the aging associated RSFC trajectories vary as a function of network definition. In line with our hypotheses, we revealed that the spatial overlap is higher in primary processing networks and if one compares different variants of the same atlas as opposed to cross-atlas comparisons. Importantly, for some brain networks effects of age and aging trajectories were dependent on atlas choice.

### 4.1 Higher-order vs. primary processing networks

The comparisons of spatial overlap revealed a non-perfect equivalence of the network definition. The overlap of higher-order networks was significantly smaller than the overlap of the primary processing networks, which is in line with a previously published study (Doucet et al., 2019). One explanation could be the oversimplistic assumption of spatially independent and/or temporally static networks, which is assumed when working with deterministic atlases (i.e., atlases in which each voxel is assigned only one value). For example, when using a probabilistic approach, it was found that 44% of the nodes are involved in more than one network, with higher-order networks having an above average number of nodes involved in multiple networks (up to more than 80%) (Yeo et al., 2014). This could indicate that spatial independence is particularly violated in brain regions of higher-order networks, which can then lead to variability in the atlases. It is also possible that these nodes do not belong to multiple networks simultaneously, but dynamically switch within a short period of time. The perspective of such dynamic connectivity is captured by the term chronnectome (Calhoun et al., 2014). It implies a model in which node involvement and connectivity patterns change within a short period of time (within several seconds), such that brain networks and/or nodes can temporarily merge and separate, reflecting dynamic segregation and integration (Iraji et al., 2019). Previous results indicate that the nodes of higher-order networks tend to have higher flexibility, that is, they tend to interact more often with other networks than nodes of primary processing networks (Cohen, 2018). This is especially true for nodes of the M-CIN, which have been reported to show the greatest flexibility (Chen et al., 2016). Consequently, the higher occurrence of these short-term RSFC changes in higher-order networks could be a cause of the lower spatial overlap in atlases.

However, even under the assumption that networks within individuals are spatially and temporally stable, the variability between individuals can influence the accuracy of the network definition. For example,Mueller et al. (2013)have shown that inter-individual variability in functional connectivity is heterogeneous across the cortex, with larger variability in higher-order networks than in primary processing networks. The variability was significantly correlated with the evolutionary expansion of the cortex, indicating an evolutive cause of variability (Buckner & Krienen, 2013;Mueller et al., 2013). It was further shown that brain regions predictive of cognitive functioning were mainly regions with high interindividual variability in connectivity (Mueller et al., 2013). In addition to variability in functional connectivity, interindividual spatial variability may also explain differences in the overlap of the networks.Laumann et al. (2015)measured an individual subject 84 times to create an individual map of resting-state networks, which was then compared to an atlas created based on 120 subjects. Although most of the networks in the atlas were included in the individual map, the authors found that the brain regions of the individual-specific networks differed in size (i.e., areas of a network occupy more/less cortex in the individual vs. in the atlas) and network membership (i.e., certain brain areas belong to a different network in the individual than in the atlas). Further, smaller subsystems/networks were found in the individual that were not included in the atlas (Laumann et al., 2015). These results were later replicated by comparing several individual-specific maps with a group map, showing that the same brain region can belong to different networks depending on the individual, especially regions near the boundaries between networks (Gordon et al., 2017).

In summary, intra- and inter-individual variability appears to be unevenly distributed across the brain, which may explain our results. Therefore, the analyses support our hypothesis of lower spatial overlap in the higher-order networks.

### 4.2 Inter-atlas differences of networks

We further found significantly smaller inter-atlas overlaps, which may indicate systematic differences in network definition between the atlases. There are three main aspects to consider when interpreting this result. First, these differences may reflect real age effects in the network architecture, as the underlying population used for atlas creation differs in age (age range: YK = 18-35, A55 = 55-95). Indeed,Doucet et al. (2021)reported differences in spatial organization between the older cohorts and the younger cohort when creating the A55, again showing less spatial agreement in higher-order networks. For example, the posterior medial temporal regions in the A55 were assigned to the L-FPN, whereas in the younger cohort they were part of the M-FPN. According to the authors, this finding supports the default to executive coupling hypothesis (DECHA) (Turner & Spreng, 2015). The idea is that the change in cognitive architecture is reflected in the architecture of the networks. That is, the age-related shift from fluid abilities to crystalline abilities could be explained by a reduction in the suppression of M-FPN activity during cognitive tasks and consequently the increase in connectivity between L-FPN and M-FPN (Spreng & Turner, 2019). However, there are further differences, for example, the superior temporal lobe was assigned to the PN in the younger cohort, whereas it was assigned to the M-FPN in the older cohort. The implications of these differences are difficult to interpret at this stage, but they should certainly be further investigated and replicated in the future. Second, parts of the differences are due to the brain areas involved for network construction. WhileYeo et al. (2011)only considered cortical regions for the definition of the networks,Doucet et al. (2021)also included subcortical areas and the cerebellum. Thus, all five networks in A55 contain subcortical and/or cerebellar brain structures that are not included in the YK. Whether or not subcortical and cerebellar areas should be included is still under debate. However, the corticocentric bias in cognitive neuroscience might be problematic (Parvizi, 2009), as cortical, subcortical, and cerebellar systems are not completely different with respect to evolution, development, or function (Chin et al., 2023). In fact, there is convincing evidence that subcortical and/or cerebellar regions are involved in higher cognitive functions, such as language (Janacsek et al., 2022), and the posterior lobe of the cerebellum reveals distinct patterns of activation depending on the cognitive task applied (Schmahmann, 2019;Stoodley & Schmahmann, 2018). Further, age-related increases in functional connectivity between cerebellar and cortical regions have already been reported (Doucet et al., 2021;Zhang et al., 2017), and this higher integration of cerebellar regions in cortical networks have been linked to successful working memory processes in the elderly population (Luis et al., 2015). Therefore, the inclusion of subcortical and cerebellar regions could be meaningful and should be included in future studies if possible. Last, there are various degrees of freedom regarding the methodological rationale when creating an atlas. These degrees of freedom include the selection of the population of interest, the measurement of the resting-state itself (eyes open vs. eyes closed) (Patriat et al., 2013), the preprocessing of the imaging data (Ciric et al., 2017), and the algorithm used to extract the networks (e.g., independent component analysis vs. clustering) (Khosla et al., 2019). Several findings suggest that these degrees of freedom can be influential. For instance, comparisons of the five networks between the three older cohorts used to create the A55 show an average overlap of 67%, with the M-CIN (46%) showing the lowest and the ON (83%) the largest overlap (Doucet et al., 2021). This is surprising considering that the age ranges of the cohorts are very similar and that the preprocessing of the data as well as the algorithm to extract the networks were identical. Consequently, the spatial differences must be attributed to differences in data collection and other network-relevant characteristics of the cohorts besides age. Indeed, the data of the three cohorts differ, for example, regarding the duration of the rs-fMRI acquisition, the voxel size, and the measurement paradigm (eyes closed vs. eyes open), which may contribute to spatial variability of the network solution. It is difficult to determine how much these individual factors should be weighted, but they do seem to be relevant, as even the ON, a well-defined primary sensory processing network, merely achieves a DSC of 0.83 due to these factors. Further evidence for the influence of methodological degrees of freedom is provided by the study ofDoucet et al. (2019), in which the overlap of networks from different atlases was calculated. This demonstrated that the DSC between networks identified based on a training sample of younger participants of a similar age range is smaller than the DSC we report here (YK vs. A55). Explicitly, the comparison of the networks by YK (age range: 18-35 years) with the networks defined in the Gordon atlas (age range: 18-33 years) (Gordon et al., 2016) yields the following DSC values: 0.56 for the ON, 0.57 for PN, 0.42 for the M-FPN, 0.50 for the L-FPN, and 0.46 for the M-CIN. Comparisons of YK with the Shirer atlas (age range: 18–30 years) (Shirer et al., 2012) revealed even smaller DSCs with 0.25 for the ON, 0.29 for PN, 0.35 for the M-FPN, 0.36 for the L-FPN, and 0.36 for the M-CIN. Consequently, the networks of YK and A55 seem to be more similar despite the age of the population used to create those two atlases being different.

Overall, the results are in line with our hypothesis: different variants of the same atlas are more similar than variants of different atlases. However, it seems that this is not mainly due to age (or other characteristics of the samples) but rather due to methodological degrees of freedom in the rs-fMRI acquisition and extraction of the networks.

### 4.3 Trajectories & age effects

For L-FPN and M-CIN, the model selection process was affected by atlas choice (i.e., linear vs. quadratic slope). Those two networks also showed the smallest spatial overlap across atlases, indicating an essential influence of network definition on RSFC change trajectories. Furthermore, despite convergent model selection, the analyses revealed a systematic difference in slope estimation between two atlases for the M-FPN. While a linear RSFC decrease was detected based on YK-N17-300, a non-significant increase over time was detected with A55-N5. While this finding is of concern, our analyses generally showed overlapping confidence intervals of the change trajectories for all other networks, thus, suggesting that atlas choice primarily changes the p-value without actually affecting the conclusion. A similar result emerged for the age effects. Here, too, all confidence intervals overlapped.

A closer look at the differences reveals that the solutions differ mainly between the atlases (A55 vs. YK). For instance, for the M-CIN, a linear change is shown with both variants of the A55, and a nonlinear change with the four variants of the YK. Conversely, there is a nonlinear change in the L-FPN with the two variants of the A55, and a linear change with three of the four variants of the YK. Consequently, the choice of atlas can influence the shape of the trajectories in certain networks. Furthermore, significant associations between age and RSFC are mainly found using the YK, with one exception (A55-N15 in M-FPN). This pattern could reflect the actual age effects in the network definition. As the A55 is adapted to older populations, the networks are already corrected for age and thus no more variance in connectivity can be explained by age.

In conclusion, the results support our hypothesis that atlas choice can affect age and time effects. However, as the influence may depend on the similarity of network definition, the impact of atlas choice is dependent on the network of interest. Future studies should therefore examine possible associations of networks with other measures (e.g., cognitive abilities) for atlas dependence, especially in the case of associations with higher-order networks.

### 4.4 Regional homogeneity and silhouette coefficient

Note that we interpret the ReHo values relative to the other values rather than in absolute terms (e.g., “good fit” or “insufficient fit”), as regressors such as global signal regression lower the absolute ReHo value, while it has no influence on the spatial distribution of ReHo (Qing et al., 2015). The ReHo values for the higher-order networks are significantly lower when using the A55 variants compared to the YK atlas variants, indicating that on average, the BOLD-signals within the voxels (or vertices)—that are averaged to a node—tend to be less similar in the A55. That is, the node definition seems to be better specified for the YK variants. This is in line with findings byGordon et al. (2016), where the YK atlas showed significantly higher ReHo values compared to the null models, whereas the AAL atlas did not (Gordon et al., 2016). This is not surprising, given that the AAL is defined on structural MRI data as opposed to the YK that is based on functional MRI data.

The SICO values show clear differences between the networks across the atlases, with the values for ON and PN tending to be higher than for the M-FPN, L-FPN, and M-CIN. This indicates that node allocation is on average less ambiguous for the primary processing networks. This finding is consistent with the idea that there are greater inter-individual differences for higher-order networks, such that the node assignment fits very well for some individuals, whereas for other individuals the nodes should rather be allocated to a different (higher-order) network. Despite the rather ambiguous node allocation for the higher-order networks, the pairwise comparisons of the SICO values at the baseline showed that the YK variants (especially the YK-N7-100) tend to be higher than the A55 variants. The additional analyses, in which the Schaefer nodes were assigned to the A55 networks, showed a slight improvement of ReHo and SICO in the individual A55 networks, but the majority of the values were still worse than for the YK (seeSupplementary Fig. 9).

Overall, only one age correlation was significant, that is, the ReHo values in the M-FPN when using the A55-N5. This indicates that the operationalization of the M-FPN using the A55-N5 gets slightly better for older individuals. However, the effect is small and all other correlations for the A55 (and YK) networks were not significant, both for ReHo and SICO. Consequently, there is no clear pattern that the atlas fit to the data improves as a function of age, which would be expected at least for the A55. This null finding is consistent with the idea that the fit of the data to the atlases remains equally good or poor regardless of the age of the individuals.

Altogether, the results indicate that the two variants of the A55 fit our dataset less well than the four variants of the YK, especially when comparing higher-order networks. These results emphasize that one should not rely on simple heuristics (e.g., similar age range or frequency of use) when choosing atlases. This is especially true when taking into account that the choice of atlas may impact age and/or time effects, as shown in this study. However, this does not mean that the networks in the A55 are poorly defined or do not match the older population in general. And it is certainly important that future studies investigating the aging process in the brain continue to test different atlases (including the A55) and their fit to the data. However, it appears that the combination of the A55 networks with the AAL3 nodes is not a particularly good choice for network operationalization (especially for higher-order networks), and thus the YK networks with Schaefer nodes may be more accurate when working with samples of older individuals.

### 4.5 Pooled effects & previous research

Although it was not the main concern in this study, we decided to calculate pooled effects for the estimates to compare our results with previous publications. Note that there have been few longitudinal studies focusing on within-network functional connectivity (Chong et al., 2019;Hafkemeijer et al., 2017;Ng et al., 2016;Oschmann & Gawryluk, 2020;Staffaroni et al., 2018), and that most of these studies consider only 2-3 measurement occasions, hindering the assessment of nonlinearity within individuals. Therefore, we can only compare the age effects and the direction of change, but not the shape (linear vs. nonlinear) of the 7-year trajectories with the previous literature.

The model selection process yielded convergent results for the two primary processing networks. For the ON, a linear increase in functional connectivity over the 7 years without additional effects of age on the intercept and the linear slope was evident. For the PN, we found a quadratic decrease of functional connectivity over the 7 years with a significant negative effect of age on the baseline RSFC. Previous longitudinal studies reported no changes in primary processing networks in older individuals (Chong et al., 2019;Hafkemeijer et al., 2017); however, these studies only examined a 2-year time span. Previous cross-sectional results on age differences for primary processing networks show considerable variability: Although the majority of studies show a reduction of functional connectivity, around 42% of studies on ON, and 21% of studies on PN show no change or an increase in connectivity with age (Deery et al., 2023). One reason for these discrepancies may be rooted in the different methodological approaches. Some studies conduct group comparisons (younger vs. older), while others treat age as a continuous variable, with the age range varying across studies. However, the study byZonneveld et al. (2019)andStumme et al. (2020)included cognitively healthy older adults in a similar age range and yet came to different results. WhileStumme and colleagues (2020)found a decrease of functional connectivity in the ON with age,Zonneveld and colleagues (2019)reported increases. This suggests that factors other than age may be relevant and perhaps systematic differences in the samples studied may result in increased or decreased RSFC being reported. Indeed, it was shown that increased white matter hyperintensities (WMH) volume was associated with increased within-network connectivity in ON, whereas the other networks showed decreased within-network connectivity with increased WMH volume (with the exception of L-FPN, which showed no association) (Kantarovich et al., 2022). WMH are strongly associated with age (Brickman et al., 2008;Chowdhury et al., 2011) and can be detected in up to 90% of MRI scans of people over 65 years of age (Schmidt et al., 2016), making WMH a relevant influencing factor in studies with older adults. However, this is only one possible factor among many, and no mechanism is known so far that specifically explains increased connectivity in ON resulting from a larger WMH volume. It is therefore not yet possible to conclusively answer this question, but our results add to the literature and indicate a linear increase in functional connectivity in the ON and a quadratic decrease in functional connectivity in the PN over a time span of 7 years.

We further estimated the pooled effects for the M-FPN, revealing a non-significant linear decrease during the 7 years with a significant negative age effect on baseline RSFC. Although not significant, the decrease of functional connectivity over time is in line with recent longitudinal studies, covering a time span of 2 to 4 years (Hafkemeijer et al., 2017;Ng et al., 2016;Oschmann & Gawryluk, 2020;Staffaroni et al., 2018), with only two reporting a significant decline (Ng et al., 2016;Staffaroni et al., 2018). Similarly, the negative age effect is in line with previous cross-sectional publications, with about 96% reporting decreases with age and no study yet reporting an increase (Deery et al., 2023).

As there was no convergence between the atlases for the trajectories in the L-FPN and M-CIN, we did not evaluate the pooled trajectories. However, for both networks, we can derive the most plausible trajectories based on the ReHo and SICO values. For the L-FPN, the highest ReHo and SICO values were achieved using the YK-N7-100, outperforming the other atlases in terms of pairwise comparisons. As seen in the model selection, using the YK-N7-100, a non-significant quadratic change over time is the most likely. Similarly, all four YK variants outperformed the A55 variants in the pairwise comparisons of the ReHo and SICO values. Using any of the four YK variants, a non-significant quadratic change over time is the most likely for the M-CIN.

The pooled age effects were negatively associated with functional connectivity at baseline, with the effect only becoming significant in the M-CIN. This suggests that functional connectivity in these two networks tends to be lower in older participants, which is consistent with previous research, pointing to decreases in functional connecitivty with higher age in about 91% of the published studies (Deery et al., 2023). However, there are some exceptions. For example,Jockwitz et al. (2017)studied a sample with an age range of 55-85 years and reported an age-related increase in functional connectivity in three higher-order networks. According to the authors, such increases could be an indication of compensation mechanisms (Jockwitz et al., 2017). It is therefore important to further explore age and time effects in order to better understand the conditions under which increases or decreases are observed, as well as the potential behavioral consequences (e.g., on cognitive abilities).

### 4.6 Strengths and limitation

A major strength of this study is the longitudinal design over 7 years comprising five measurement time points. This allowed us to estimate the influence of atlas choice on real change over time and especially on the shape of trajectories. Previous longitudinal studies on functional connectivity in aging research are very rare and limited to two to three measurement occasions, making nonlinear trajectories within individuals difficult to demonstrate. Another strength of the study is the use of different variants of the same atlas. Thus, we were able to show that the networks are not only different between the two atlases, but also that there is no perfect spatial overlap between the variants of the atlases (e.g., 5 vs. 15 networks). Consequently, there may be partially different results with regard to age and time effects, depending on which variant is chosen.

There are also limitations in this study that should be mentioned. These mainly concern methodological aspects. First, there are many degrees of freedom in the methodological decisions, such as the preprocessing of the data or the choice of quality criteria for the model selection. Also, the selection of nodes in terms of type (functional or structural) and number to compute connectivity within networks is partially arbitrary, which is a fundamental problem in network neuroscience (Zalesky et al., 2010). Hence, we cannot generalize our results across different preprocessing pipelines, statistical methods, and network constructions. Second, we have only examined two atlases here, so we cannot determine whether the use of other atlases and their variants would have yielded similar results.

### 4.7 Practical implications and future directions

Overall, based on the literature and our reported findings, we recommend that previous results should be interpreted with caution and that future studies should increase their focus on atlas selection and its justification.

More specifically, first, it would be desirable to use more than one atlas when specifically studying networks, so that replicability between atlases can be assessed and pooled effects at the network level can be calculated. In order to guide the decision process for the primary atlas used in a study or to assess the quality of the results, determining how well an atlas fits the actual data may be helpful. To do so, we recommend determining the regional homogeneity of the nodes to evaluate which atlas best merges the BOLD signals at the voxel or vertices level. In a second step, the allocation of the nodes to the networks can be calculated, for example, using silhouette coefficients. This quality control procedure seems particularly important when studying higher-order networks, as higher-order networks have greater variability between atlases (less spatial overlap) and between individuals. If two atlases perform equally well, the frequency of atlas use could serve as a further decision criterion, as this increases comparability with previous studies. To limit arbitrary decisions in the use of atlases and the methodological approach in general, we recommend to pre-register the intended study design.

Second, to better understand demographic effects such as age on network definition, we would like to encourage authors who create an atlas to make it publicly available. In doing so, it would be desirable that networks are ideally subdivided into meaningful nodes, preferably based on fMRI resting state data.

Third, in order to use intra- and inter-network metrics as biomarkers in the long term, it would be beneficial to identify which network definitions can make particularly accurate predictions, for example, regarding cognitive abilities or the development of dementia.

Above all, more longitudinal studies are needed to better understand changes of functional brain network characteristics within the individual.

## 5 Conclusion

The atlas choice affects the estimated average functional connectivity in various networks, which highlights the importance of this methodological decision for future studies and calls for careful interpretation of already published results. Ultimately, there is no standard about how to operationalize networks. However, future studies may use and compare multiple atlases to assess the impact of network definition on outcomes. Furthermore, the validity and predictive power of specific network definitions could be assessed by calculating their associations with behavioral outcomes (e.g., cognitive ability).

## Supplementary Material

Supplementary Material

## Data Availability

The data supporting this manuscript are not publicly available because the used consent does not allow for the public sharing of the data.

## Software Availability Statement

The following openly available software were used:

fMRIPrep (https://fmriprep.org/en/stable/)

R (https://www.r-project.org/)
