# Supplementary material for: Influence of atlas-choice on age and time effects in large-scale brain networks in the context of healthy aging
Source: Imaging Neurosci (Camb). 2024 Apr 8;2:imag-2-00127. doi: 10.1162/imag_a_00127 (PMC12247562; doi:10.1162/imag_a_00127)
Supplement: Supplementary Material [file imag_a_00127-supp.pdf]

Supplementary Information

Supplementary Text 1

Literature Screening

The platform Pubmed (https://pubmed.ncbi.nlm.nih.gov/) was used for the screening of the literature. The search terms "resting-state networks" and "healthy aging" were entered and the age filter "65+ years" was activated. This resulted in a list of 284 studies (as of end of June 2022) published between 2004 and 2022. Of the 284 studies, 45 (15.85%) were excluded. The reasons were as follows: no fMRI used (n = 38), review article (n=1), animal study (n=1), no focus on healthy aging (n=3), or age of sample less than 65 years (n=1), no access to article (n=1).

Of the total 239 included articles, 87 (36.4%) applied the seed approach, 57 (23.8%) applied Independent Component Analysis (ICA), 36 (15.1%) used graph-theoretic approaches, and 57 (23.8%) reported multiple approaches (e.g., ICA and graph analysis). Two (0.8%) were classified as “other”. In these studies, other approaches were used, such as spatiotemporal frequency domain analyses.

Atlases were mentioned 115 times in the studies, either to divide the brain into nodes/parcels, or to extract networks. The most used templates for parceling the brain into nodes was the Automatic Anatomical Labeling (AAL) atlas (Tzourio-Mazoyer et al., 2002). For network extraction, the most used template was the Yeo-Krienen atlas (Yeo et al., 2011).

Supplementary Figure 1

Count of Atlases used for Network Definition Based on Literature Screening

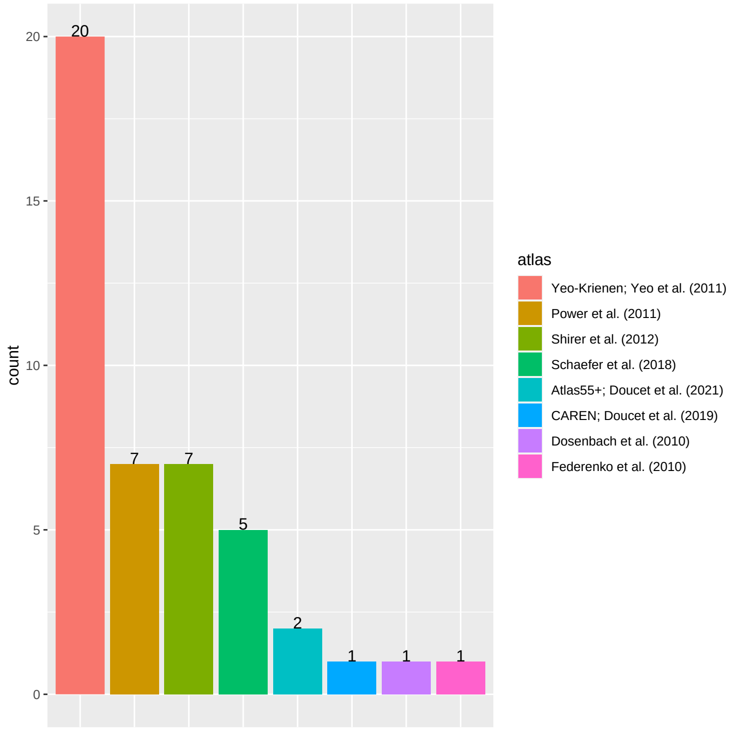

Supplementary Figure 2

Count of Atlases used for Node Definition Based on Literature Screening

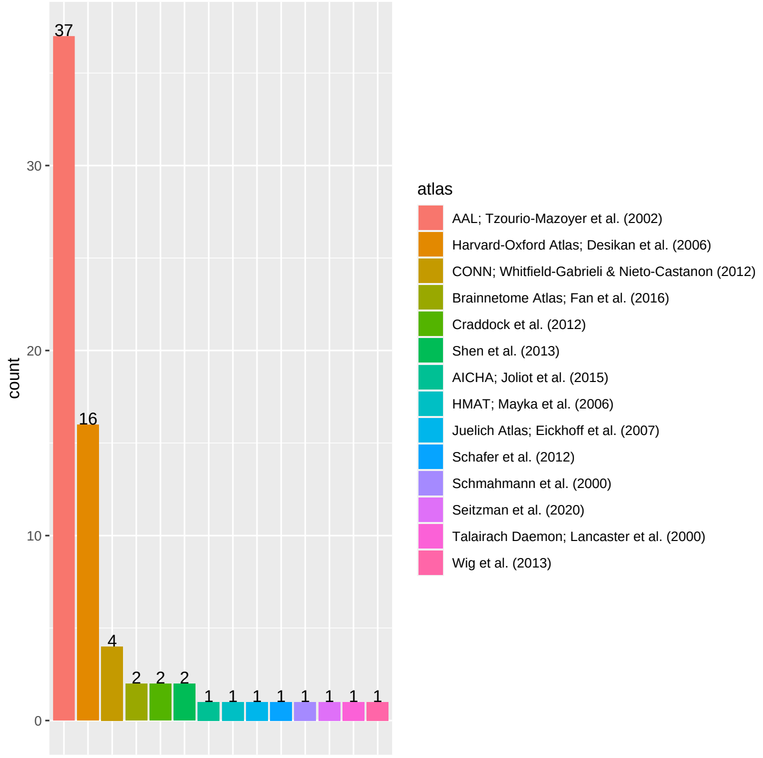

Supplementary Figure 3

Network Resolution and Number of Nodes Used for Yeo-Krienen Atlas in Literature Screening

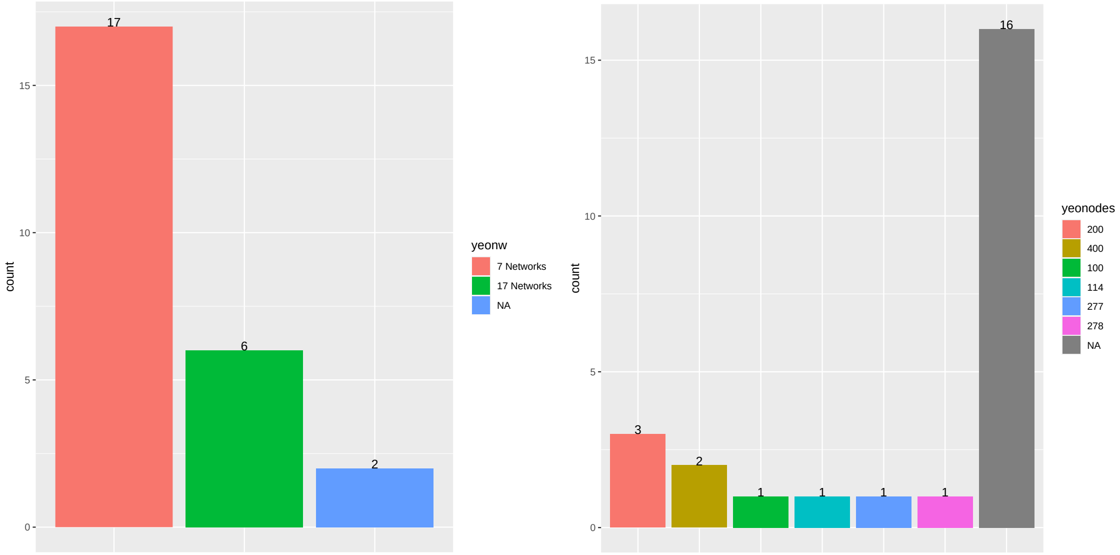

Abbreviations: yeonw = Number of networks (resolution) used within Yeo-Krienen atlas; yeonodes = Number of nodes used within Yeo-Krienen atlas.

Supplementary Figure 4

Exemplary Visualization of the Occipital Subnetworks by Atlas.

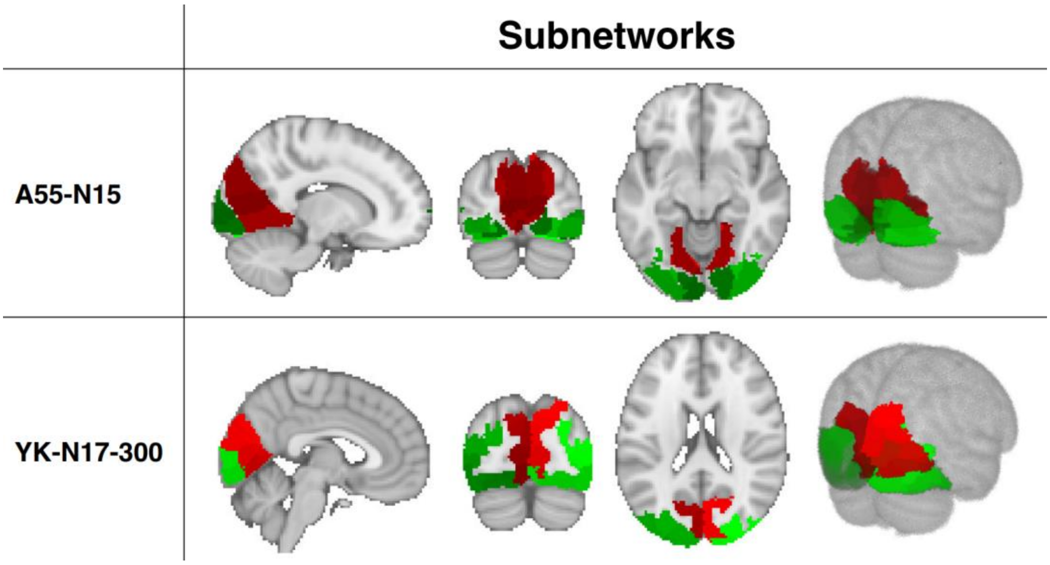

Note. Red and green regions represent the two subnetworks of the occipital network in each of the two atlases (Atlas55+ with 15 networks and Yeo-Krienen atlas with 17 networks). For A55-N15, the red colored brain regions correspond to the medial visual subnetwork and the green colored brain regions to the posterior visual subnetwork. For YK-N17-300, the red colored brain regions correspond to the central visual subnetwork and the green colored brain regions to the peripheral visual subnetwork. The different color shades correspond to the nodes (for YK the Schaefer nodes and for A55 the AAL nodes).

Supplementary Figure 5

Exemplary Visualization of the Medial Frontoparietal Network of the Atlas55+ with Schaefer Nodes

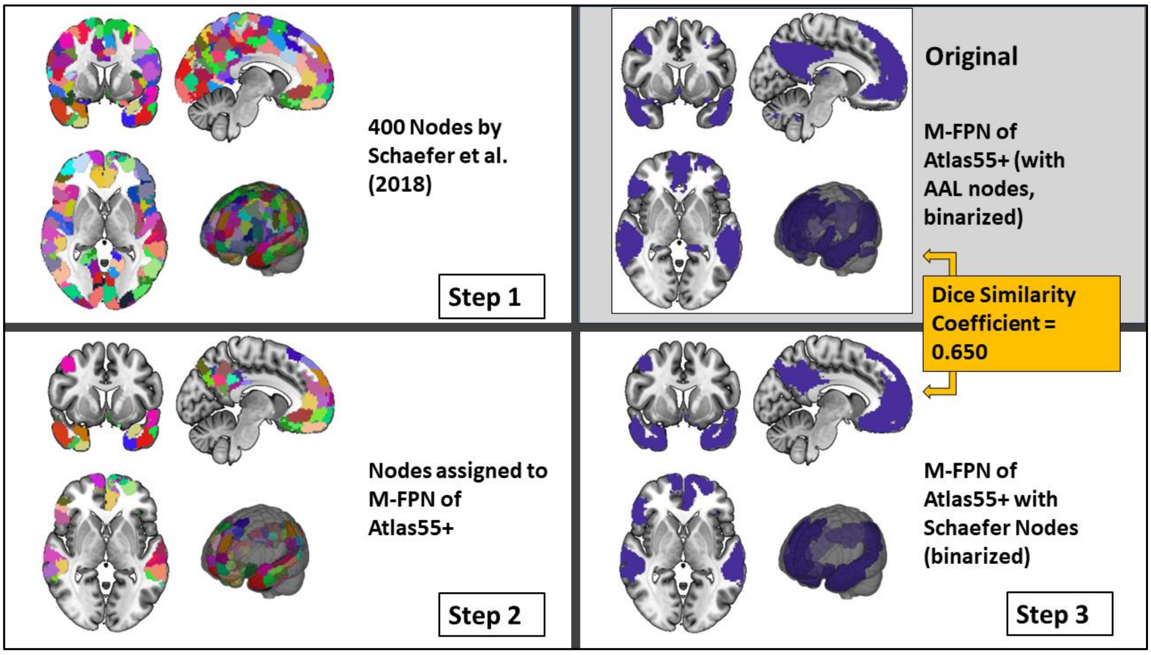

Note. For a full list of dice similarity coefficient between the original networks as defined in Atlas55+ and the networks defined using nodes by Schaefer et al. (2018), see Supplementary Table 7 below.

Supplementary Figure 6

Dice Similarity Coefficient Between each Atlas and Variant Thereof for each Network

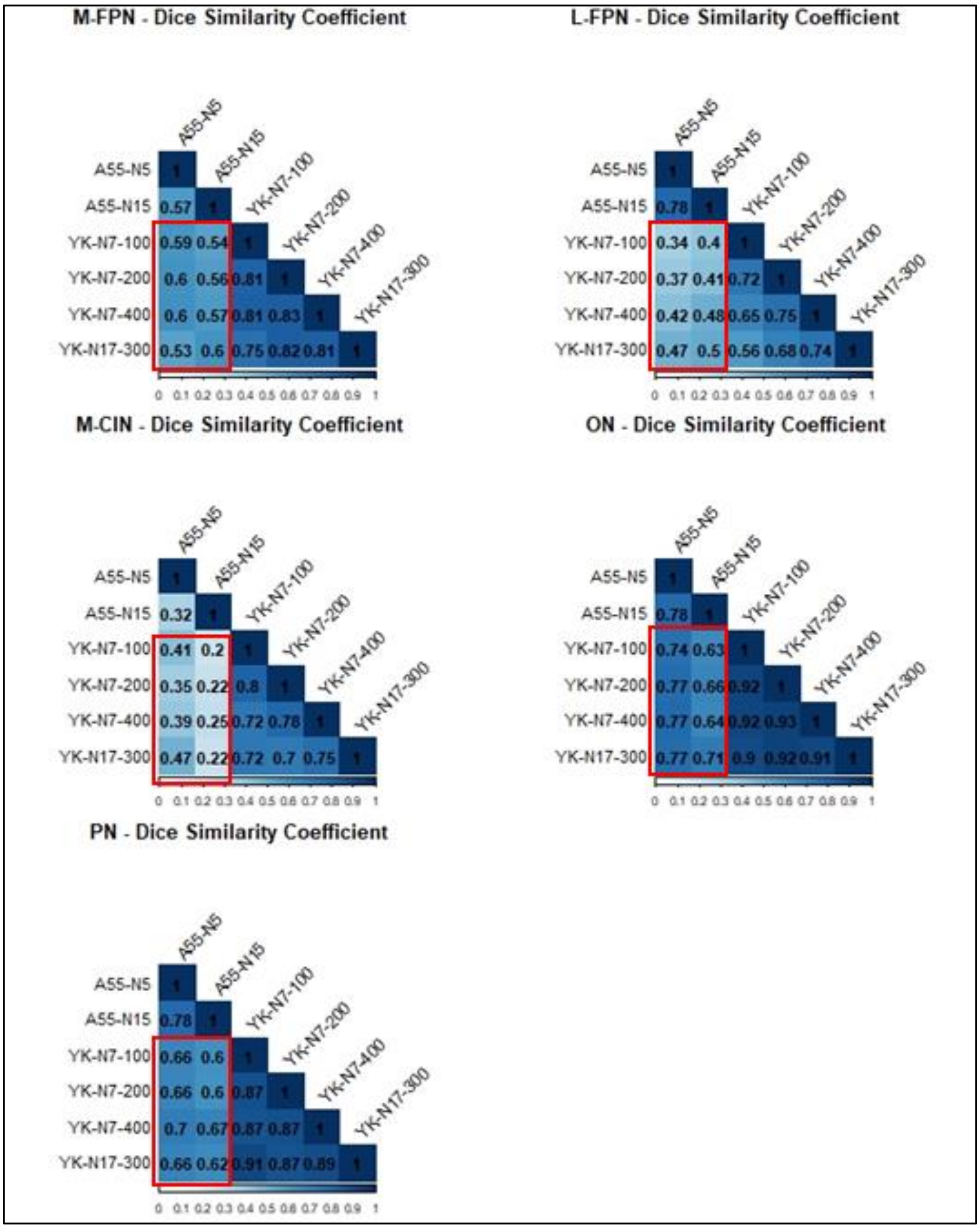

Note. Values in the red box correspond to the overlap between the atlases (inter-atlas), while the other values show the overlap within the atlases (i.e., intra-atlas = between variants of the same atlas).

Supplementary Figure 7

Summary of the Estimates for Sex Effects and their Confidence Intervals for each Atlas and Variant Used

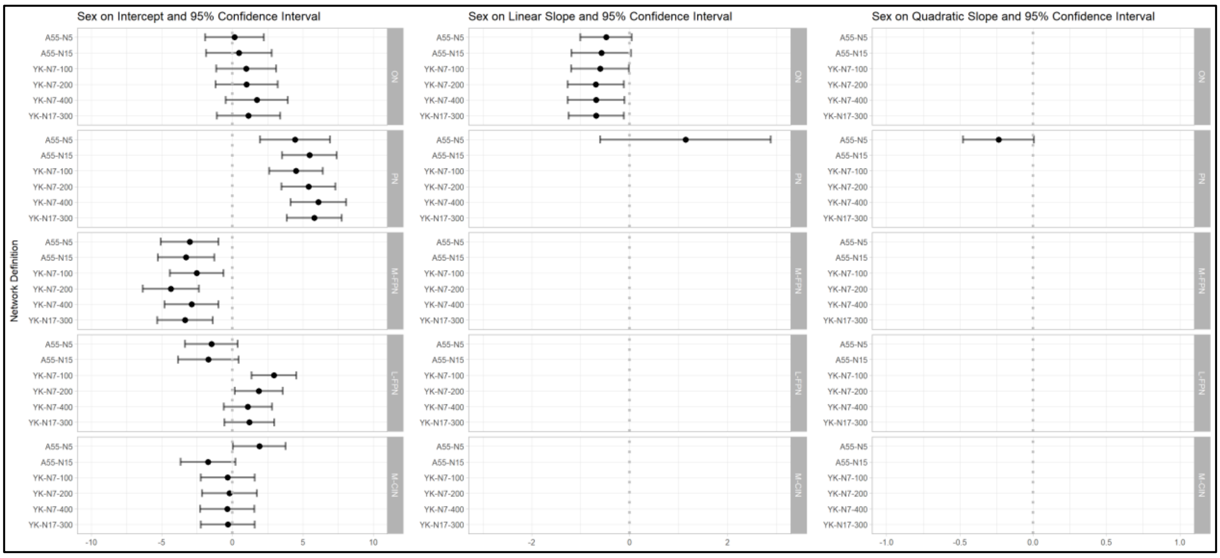

Note. Confidence intervals that cross the dotted line indicate no statistical significance. The unit of the effects corresponds to t-scores (10 equals one standard deviation). Sex was coded as female = 0 and male = 1 (e.g., men show higher RSFC at baseline in the PN and lower RSFC in the M-FPN).

Supplementary Figure 8

Summary of the Estimates for Education Effects and their Confidence Intervals for each Atlas and Variant Used

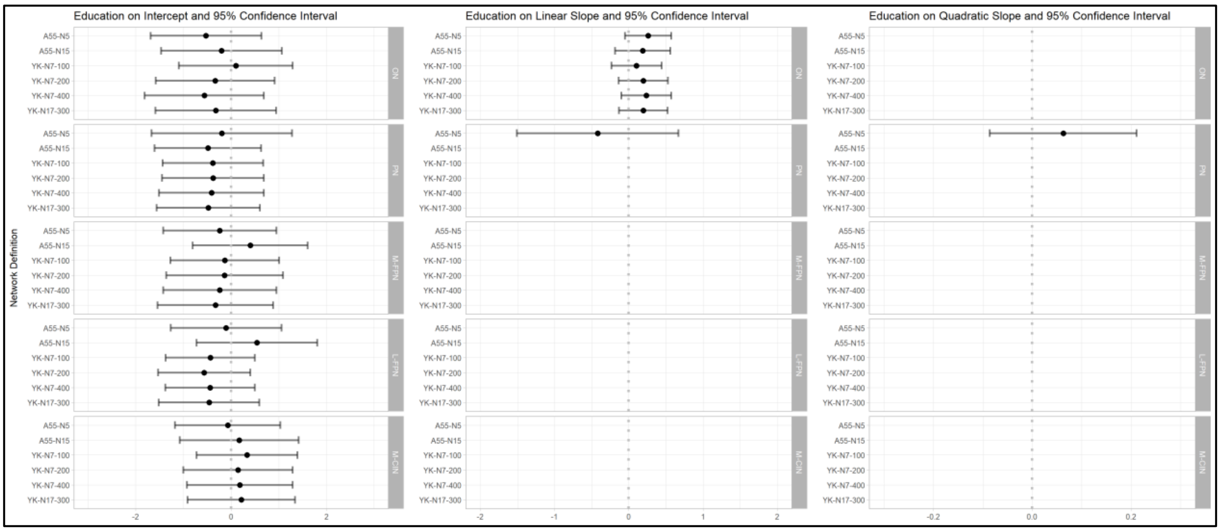

Note. Confidence intervals that cross the dotted line indicate no statistical significance. The unit of the effects corresponds to t-scores (10 equals one standard deviation). Education was codes as follows: -1 = high school with or without vocational education; 0 = higher education entrance qualification, business school or university of applied sciences; 1 = university degree). There are no significant effects of education for any atlas used.

Supplementary Figure 9

Regional Homogeneity and Silhouette Coefficient for each Network and Atlas with Bonferroni Corrected Results of Wilcoxon-Test between Atlases.

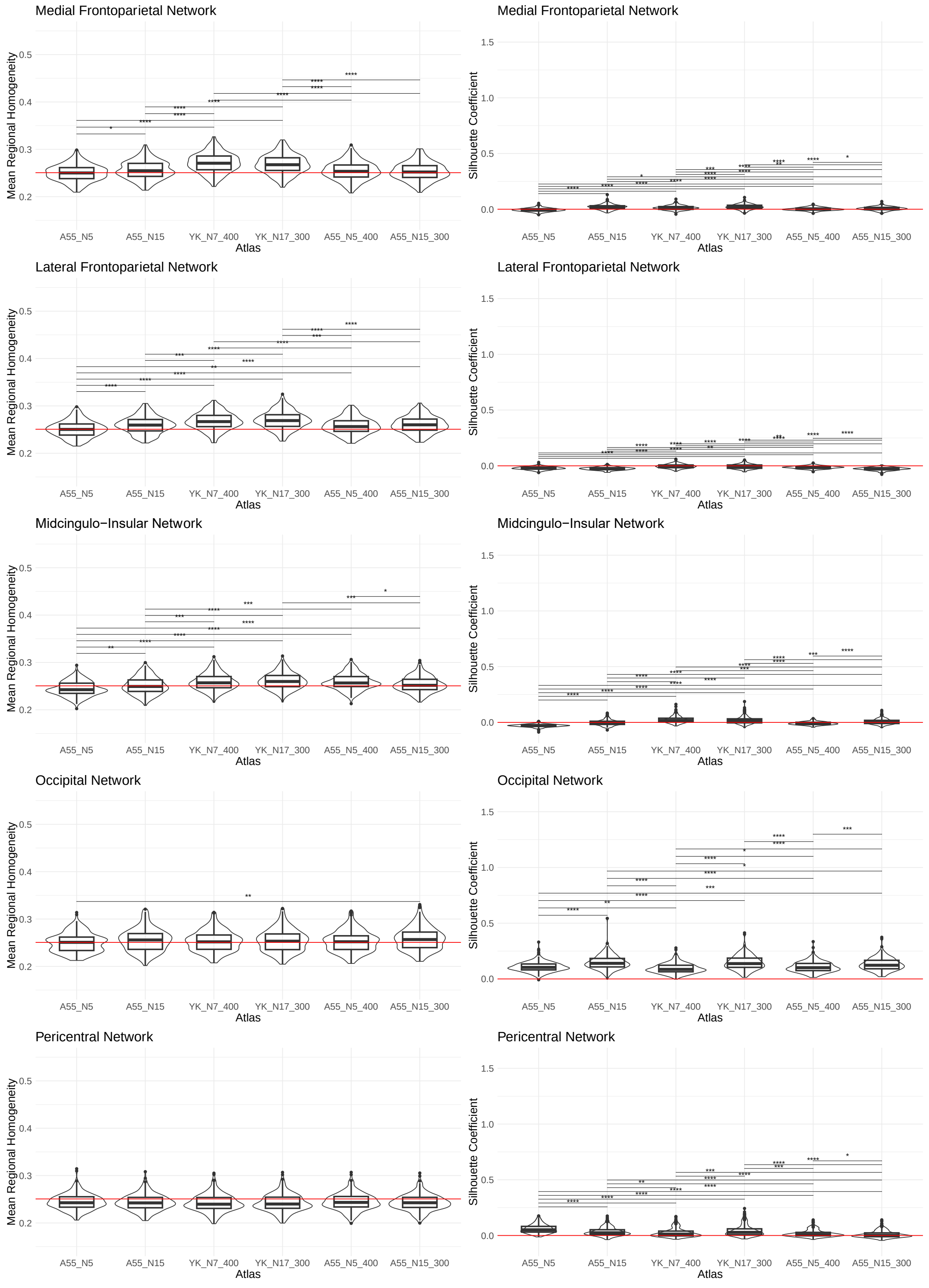

Abbreviations: A55 = Atlas55+; ON = Occipital Network; PN = Pericentral Network; M-FPN = Meidal Frontoparietal Network; L-FPN = Lateral Frontoparietal Network; M-CIN = Midcingulo-Insular Network

Note. A55-N5 and A55-N15 correspond to the original Atlas55+ and were analyzed using nodes of the automated anatomical labeling atlas. A55-N5-400 and A55-N15-300 correspond to the networks when

assigning the 400 or 300 Schaefers node to the networks by Atlas55+ that show the highest overlap. A more detailed description of the node assignment to the A55 network can be found in the main text post-hoc analysis of atlas fit. The red lines in the left plots (ReHo) indicate the average ReHo value across the cortex (*M* = 0.251). The red lines in the right plots (SICO) are placed at 0. Values above the line indicate an acceptable allocation of the nodes to the networks.

Supplementary Table 1

Frequently used Atlases for Node and/or Network Definition with Sample Size and Age Range of the Underlying Population.

Atlas	Sample Size	Age Range in Years
Tzourio-Mazoyer et al. (2002), Rolls et al. (2015), Rolls et al. (2020), AAL1-3	N = 1 (repeatedly measured, n = 27)	not stated ("young healthy")
Desikan et al. (2006), Harvard-Oxford atlas	N = 40	19-86
Smith et al. (2009)*	N = 36	20-35
Yeo et al. (2011)*	n_1 = 500;<br>n_2 = 500 (replication)	18-35
Doucet et al. (2011)*	N = 180	18-57
Power et al. (2011)	n_1 > 300; n_2 = 40; n_3 = 106	7-30
Shirer et al. (2012)*	N = 15	18-30
Craddock et al. (2012)	N = 41	18-55
Shen et al. (2013)	N = 79	not stated
Fan et al. (2016), Brainnetome	n_1 = 40<br>n_2 = 40 (replication)	22-35<br>17-20
Glasser et al. (2016)	n_1 = 210;<br>n_2 = 201 (replication)	22-35
Gordon et al. (2016)*	N = 108	18-33
Doucet, Rasgon, et al. (2018)*	N = 496	22-37
Schaefer et al. (2018)	N = 1489	18-35
Doucet et al. (2019), CAREN	combination of four atlases	18-57
Ji et al. (2019)	N = 337	22-37
Urchs et al. (2019)	N = 198	18-30
Doucet et al. (2021), Atlas 55+	N = 563	55-95

Note. The spatial overlaps of the networks from the atlases marked with an asterisk were investigated by Doucet et al. (2019).

Supplementary Table 2

Participant Characteristics at Baseline of the Full Sample and at Each Follow-up Wave.

Variable	Baseline (*n* = 232)			1-y follow-up (*n* = 211)			2-y follow-up (n = 197)			4-y follow-up (*n* = 173)			7-y follow-up (*n* = 125)			Total selectivity
	n	M	SD	*n*	M	SD	*n*	M	SD	*n*	M	SD	n	M	SD	
Age (years)	232	70.85	5.09	211	70.95	5.15	197	70.67	4.81	173	70.16	4.44	125	70.03	4.07	-0.16
Gender (% f)	232	49.1	-	211	48.3	-	197	46.7	-	173	46.2	-	125	40	-	-
Education (1-3)	226	2.23	0.86	211	2.24	0.86	196	2.22	0.87	172	2.28	0.85	125	2.36	0.82	0.16
Mental health	212	54.80	6.22	195	54.62	6.35	184	54.56	6.20	159	54.70	5.67	116	54.95	5.40	0.03
Physical health	212	50.80	7.44	195	50.91	7.45	184	51.05	6.96	159	51.44	6.45	116	51.08	6.98	0.04
MMSE	232	28.83	1.02	211	28.86	1.00	197	28.87	1.00	173	28.90	0.98	125	28.90	0.94	0.07

Note. f = female. Education was measured on a scale from 1 to 3 (1 = high school with or without vocational education, 2 = higher education entrance qualification, business school or university of applied sciences, or 3 = university degree). Mental and physical health scores were computed based on the SF12 questionnaire, which participants filled out at home (Ware, Kosinski, & Keller, 1996). Total selectivity was computed for the baseline sample as compared to baseline values of the remaining sample at 7-y follow-up $(M_{7\text{-}y} - M_{base})/SD_{base}$).

Supplementary Table 3

Model Fit Critera for each Network and Model by Atlas and Variants

Medial Frontoparietal Network

	**Model 1**		**Model 2**		**Model 3**		**Model 4**		**Model 5**	
	**Est**	**Int**	**Est**	**Int**	**Est**	**Int**	**Est**	**Int**	**Est**	**Int**
**A55-N5**										
***X*²/df**	5.304	poor	1.3	good	1.359	good	1.235	good	1.213	good
***p*-value**	0	poor	0.133	good	0.116	good	0.186	good	0.248	good
**CFI**	0.033	poor	0.954	accept	0.955	accept	0.968	accept	0.988	good
**RMSEA**	0.137	poor	0.032	good	0.035	good	0.027	good	0.022	good
**BIC**	8910	5	8799	1	8821	3	8801	2	8847	4
**Heywood**	0	no	0	no	1	yes	0	no	1	Yes
**A55-N15**										
***X*²/df**	7.791	poor	0.968	good	0.822	good	0.934	good	0.88	good
***p*-value**	0	poor	0.513	good	0.706	good	0.563	good	0.593	good
**CFI**	0.034	poor	1	good	1	good	1	good	1	good

**RMSEA**	0.172	poor	0	good	0	good	0	good	0	good
**BIC**	8779	5	8579	1	8598	3	8582	2	8631	4
**Heywood**	0	no	0	no	1	yes	0	no	1	yes
**YK-N7-100**										
***X*²/df**	6.029	poor	1.424	good	1.412	good	1.422	good	1.222	good
***p*-value**	0	poor	0.068	good	0.09	good	0.072	good	0.241	good
**CFI**	0.057	poor	0.93	poor	0.944	poor	0.932	poor	0.976	good
**RMSEA**	0.149	poor	0.043	good	0.043	good	0.044	good	0.034	good
**BIC**	8751	5	8620	1	8639	3	8624	2	8665	4
**Heywood**	0	no	0	no	1	yes	0	no	1	yes
**YK-N7-200**										
***X*²/df**	7.279	poor	1.158	good	1.211	good	1.126	good	1.307	good
***p*-value**	0	poor	0.257	good	0.221	good	0.297	good	0.182	good
**CFI**	0.021	poor	0.983	good	0.981	good	0.988	good	0.978	good
**RMSEA**	0.166	poor	0.023	good	0.027	good	0.02	good	0.035	good
**BIC**	8872	5	8693	1	8716	3	8697	2	8747	4
**Heywood**	0	no	0	no	1	yes	0	no	1	yes
**YK-N7-400**										
***X*²/df**	6.331	poor	1.234	good	1.212	good	1.223	good	1.158	good
***p*-value**	0	poor	0.183	good	0.221	good	0.197	good	0.294	good
**CFI**	0.059	poor	0.967	accept	0.977	good	0.969	accept	0.988	good
**RMSEA**	0.153	poor	0.031	good	0.028	good	0.03	good	0.024	good
**BIC**	8787	5	8641	1	8662	3	8645	2	8690	4
**Heywood**	0	no	0	no	1	yes	0	no	1	yes
**YK-N17-300**										
***X*²/df**	7.123	poor	1.145	good	1.148	good	1.026	good	1.069	good
***p*-value**	0	poor	0.272	good	0.283	good	0.427	good	0.379	good
**CFI**	0.035	poor	0.992	good	0.994	good	1	good	1	good
**RMSEA**	0.164	poor	0.016	good	0.015	good	0	good	0	good
**BIC**	8815	5	8641	1	8662	3	8642	2	8691	4
**Heywood**	0	no	0	no	1	yes	0	no	1	yes

Lateral Frontoparietal Network

	**Model 1**		**Model 2**		**Model 3**		**Model 4**		**Model 5**	
	**Est**	**Int**	**Est**	**Int**	**Est**	**Int**	**Est**	**Int**	**Est**	**Int**
**A55-N5**										
***X*²/df**	3.288	poor	1.27	good	1.354	good	1.064	good	1.375	good

***p*-value**	0	poor	0.154	good	0.119	good	0.374	good	0.143	good
**CFI**	0.076	poor	0.982	good	0.939	poor	1	good	0.936	poor
**RMSEA**	0.096	poor	0.014	good	0.029	good	0	good	0.036	good
**BIC**	8816	5	8768	2	8791	3	8767	1	8820	4
**Heywood**	0	no	0	no	1	yes	0	no	2	yes
**A55-N15**										
X^2/df	3.753	poor	1.801	good	1.931	good	1.654	good	2.169	accept
***p*-value**	0	poor	0.006	poor	0.005	poor	0.018	accept	0.004	poor
**CFI**	0	poor	0.804	poor	0.784	poor	0.867	poor	0.799	poor
**RMSEA**	0.105	poor	0.048	good	0.056	accept	0.04	good	0.065	accept
**BIC**	8928	5	8880	2	8901	3	8879	1	8929	4
**Heywood**	0	no	0	no	1	yes	0	no	2	yes
**YK-N7-100**										
X^2/df	3.059	poor	1.099	good	1.063	good	1.006	good	0.831	good
***p*-value**	0	poor	0.327	good	0.379	good	0.455	good	0.651	good
**CFI**	0.12	poor	0.962	accept	0.981	good	0.995	good	1	good
**RMSEA**	0.096	poor	0.021	good	0.017	good	0.008	good	0	good
**BIC**	8658	4	8613	1	8634	3	8615	2	8661	5
**Heywood**	0	no	0	no	0	no	0	no	0	no
**YK-N7-200**										
X^2/df	2.866	accept	1.029	good	0.806	good	0.935	good	0.595	good
***p*-value**	0	poor	0.422	good	0.728	good	0.561	good	0.89	good
**CFI**	0.131	poor	0.992	good	1	good	1	good	1	good
**RMSEA**	0.091	poor	0.009	good	0	good	0	good	0	good
**BIC**	8704	3	8662	1	8679	3	8664	2	8708	5
**Heywood**	0	no	0	no	1	yes	0	no	3	yes
**YK-N7-400**										
X^2/df	2.813	accept	0.863	good	0.779	good	0.818	good	0.713	good
***p*-value**	0	poor	0.672	good	0.762	good	0.733	good	0.784	good
**CFI**	0.173	poor	1	good	1	good	1	good	1	good
**RMSEA**	0.089	poor	0	good	0	good	0	good	0	good
**BIC**	8694	4	8650	1	8671	3	8653	2	8702	5
**Heywood**	0	no	0	no	0	no	0	no	0	no
**YK-N17-300**										
X^2/df	2.139	accept	1.023	good	1.013	good	0.948	good	0.864	good

***p*-value**	0	poor	0.431	good	0.443	good	0.541	good	0.612	good
**CFI**	0.346	poor	1	good	1	good	1	good	1	good
**RMSEA**	0.062	accept	0	good	0	good	0	good	0	good
**BIC**	8814	3	8796	1	8818	4	8798	2	8846	5
**Heywood**	0	no	0	no	1	yes	0	no	2	yes

Midcingulo-Insular Network

	**Model 1**		**Model 2**		**Model 3**		**Model 4**		**Model 5**	
	**Est**	**Int**	**Est**	**Int**	**Est**	**Int**	**Est**	**Int**	**Est**	**Int**
**A55-N5**										
***X*²/df**	4.579	poor	1.06	good	1.202	good	0.984	good	1.186	good
***p*-value**	0	poor	0.378	good	0.229	good	0.487	good	0.27	good
**CFI**	0.055	poor	1	good	0.981	good	1	good	0.989	good
**RMSEA**	0.124	poor	0	good	0.021	good	0	good	0.019	good
**BIC**	8869	5	8774	1	8799	3	8777	2	8829	4
**Heywood**	0	no	0	no	0	no	0	no	0	no
**A55-N15**										
***X*²/df**	7.299	poor	1.451	good	1.42	good	1.39	good	1.516	good
***p*-value**	0	poor	0.058	good	0.087	good	0.085	good	0.084	good
**CFI**	0.031	poor	0.955	accept	0.962	accept	0.965	accept	0.967	accept
**RMSEA**	0.166	poor	0.038	good	0.038	good	0.034	good	0.043	good
**BIC**	8721	5	8550	1	8569	3	8552	2	8598	4
**Heywood**	0	no	0	no	1	yes	0	no	2	yes
**YK-N7-100**										
***X*²/df**	5.27	poor	1.51	good	1.346	good	1.244	good	1.12	good
***p*-value**	0	poor	0.041	accept	0.124	good	0.178	good	0.329	good
**CFI**	0.031	poor	0.901	poor	0.945	poor	0.955	accept	0.987	good
**RMSEA**	0.137	poor	0.047	good	0.039	good	0.032	good	0.022	good
**BIC**	8780	5	8676	2	8691	3	8672	1	8716	4
**Heywood**	0	no	0	no	1	yes	0	no	3	yes
**YK-N7-200**										
***X*²/df**	6.279	poor	1.582	good	1.403	good	1.288	good	1.002	good
***p*-value**	0	poor	0.026	accept	0.095	good	0.144	good	0.451	good
**CFI**	0.024	poor	0.914	poor	0.955	accept	0.963	accept	1	good
**RMSEA**	0.152	poor	0.048	good	0.039	good	0.032	good	0	good
**BIC**	8779	5	8644	2	8659	3	8640	1	8681	4
**Heywood**	0	no	0	no	1	yes	0	no	1	yes

**YK-N7-400**										
***X*²/df**	6.204	poor	1.65	good	1.589	good	1.321	good	1.244	good
***p*-value**	0	poor	0.017	accept	0.036	accept	0.123	good	0.225	good
**CFI**	0.021	poor	0.899	poor	0.925	poor	0.954	accept	0.979	good
**RMSEA**	0.151	poor	0.052	accept	0.049	good	0.036	good	0.031	good
**BIC**	8755	5	8624	2	8642	3	8619	1	8663	4
**Heywood**	0	no	0	no	1	yes	0	no	1	yes
**YK-N17-300**										
***X*²/df**	5.491	poor	1.959	good	2.068	good	1.68	good	1.856	good
***p*-value**	0	poor	0.002	poor	0.002	poor	0.015	accept	0.02	accept
**CFI**	0	poor	0.819	poor	0.832	poor	0.881	poor	0.909	poor
**RMSEA**	0.139	poor	0.062	accept	0.066	accept	0.051	accept	0.058	accept
**BIC**	8816	5	8717	2	8737	3	8713	1	8757	4
**Heywood**	0	no	0	no	1	yes	0	no	2	yes

Pericentral Network

	**Model 1**		**Model 2**		**Model 3**		**Model 4**		**Model 5**	
	**Est**	**Int**	**Est**	**Int**	**Est**	**Int**	**Est**	**Int**	**Est**	**Int**
**A55-N5**										
***X*²/df**	5.505	poor	1.371	good	1.429	good	1.402	good	1.453	good
***p*-value**	0	poor	0.091	good	0.083	good	0.08	good	0.107	good
**CFI**	0.066	poor	0.949	poor	0.944	poor	0.945	poor	0.962	accept
**RMSEA**	0.14	poor	0.035	good	0.041	good	0.037	good	0.04	good
**BIC**	8762	5	8645	1	8667	3	8650	2	8695	4
**Heywood**	0	no	0	no	1	yes	0	no	0	no
**A55-N15**										
***X*²/df**	7.261	poor	1.461	good	1.539	good	1.192	good	1.265	good
***p*-value**	0	poor	0.055	good	0.048	accept	0.226	good	0.21	good
**CFI**	0.019	poor	0.948	poor	0.947	poor	0.985	good	0.984	good
**RMSEA**	0.166	poor	0.041	good	0.046	good	0.023	good	0.03	good
**BIC**	8814	5	8645	2	8666	3	8641	1	8689	4
**Heywood**	0	no	0	no	1	yes	0	no	1	yes
**YK-N7-100**										
***X*²/df**	6.827	poor	1.376	good	1.422	good	1.182	good	1.452	good
***p*-value**	0	poor	0.089	good	0.086	good	0.236	good	0.108	good
**CFI**	0.02	poor	0.95	accept	0.955	accept	0.98	good	0.965	accept
**RMSEA**	0.16	poor	0.039	good	0.04	good	0.025	good	0.043	good

**BIC**	8813	5	8655	2	8676	3	8653	1	8705	4
**Heywood**	0	no	0	no	1	yes	0	no	2	yes
**YK-N7-200**										
***X*²/df**	8.145	poor	1.561	good	1.544	good	1.386	good	1.416	good
***p*-value**	0	poor	0.03	accept	0.046	accept	0.087	good	0.123	good
**CFI**	0.01	poor	0.938	poor	0.953	accept	0.961	accept	0.977	good
**RMSEA**	0.177	poor	0.048	good	0.046	good	0.038	good	0.038	good
**BIC**	8812	5	8616	1	8635	3	8616	1	8661	4
**Heywood**	0	no	0	no	1	yes	0	no	5	yes
**YK-N7-400**										
***X*²/df**	9.328	poor	1.879	good	1.917	good	1.61	good	1.817	good
***p*-value**	0	poor	0.003	poor	0.005	poor	0.024	accept	0.023	accept
**CFI**	0.003	poor	0.917	poor	0.93	poor	0.947	poor	0.958	accept
**RMSEA**	0.191	poor	0.059	good	0.06	good	0.048	good	0.055	accept
**BIC**	8830	5	8605	2	8624	3	8602	1	8647	4
**Heywood**	0	no	0	no	1	yes	0	no	2	yes
**YK-N17-300**										
***X*²/df**	8.692	poor	1.848	good	1.83	good	1.619	good	1.805	good
***p*-value**	0	poor	0.004	poor	0.009	poor	0.022	accept	0.025	accept
**CFI**	0.006	poor	0.911	poor	0.931	poor	0.939	poor	0.953	accept
**RMSEA**	0.184	poor	0.059	accept	0.057	accept	0.049	good	0.056	accept
**BIC**	8818	5	8613	2	8631	3	8611	1	8655	4
**Heywood**	0	no	0	no	1	yes	0	no	2	yes

Occipital Network

	**Model 1**		**Model 2**		**Model 3**		**Model 4**		**Model 5**	
	**Est**	**Int**	**Est**	**Int**	**Est**	**Int**	**Est**	**Int**	**Est**	**Int**
**A55-N5**										
***X*²/df**	3.736	poor	1.501	good	1.263	good	1.517	good	1.303	good
***p*-value**	0	poor	0.043	accept	0.179	good	0.042	accept	0.185	good
**CFI**	0.07	poor	0.869	poor	0.957	accept	0.871	poor	0.967	accept
**RMSEA**	0.109	poor	0.044	good	0.028	good	0.044	good	0.029	good
**BIC**	8612	5	8556	1	8570	3	8561	2	8600	4
**Heywood**	0	no	0	no	0	no	0	no	3	yes
**A55-N15**										
***X*²/df**	3.978	poor	1.311	good	0.978	good	1.308	good	0.913	good
***p*-value**	0	poor	0.125	good	0.491	good	0.131	good	0.553	good

**CFI**	0.082	poor	0.921	poor	1	good	0.926	poor	1	good
**RMSEA**	0.115	poor	0.036	good	0	good	0.035	good	0	good
**BIC**	8711	5	8642	1	8655	3	8646	2	8685	4
**Heywood**	0	no	0	no	0	no	0	no	3	yes
**YK-N7-100**										
***X*²/df**	4.074	poor	1.44	good	1.056	good	1.455	good	1.143	good
***p*-value**	0	poor	0.062	good	0.388	good	0.06	good	0.307	good
**CFI**	0.082	poor	0.896	poor	1	good	0.896	poor	0.988	good
**RMSEA**	0.116	poor	0.042	good	0	good	0.042	good	0.019	good
**BIC**	8664	5	8596	1	8607	3	8600	2	8639	4
**Heywood**	0	no	0	no	0	no	0	no	0	no
**YK-N7-200**										
***X*²/df**	4.538	poor	1.487	good	1.26	good	1.541	good	1.422	good
***p*-value**	0	poor	0.047	accept	0.181	good	0.036	accept	0.12	good
**CFI**	0.091	poor	0.911	poor	0.976	good	0.903	poor	0.962	accept
**RMSEA**	0.124	poor	0.041	good	0.024	good	0.044	good	0.036	good
**BIC**	8702	5	8621	1	8635	3	8626	2	8667	4
**Heywood**	0	no	0	no	0	no	0	no	4	yes
**YK-N7-400**										
***X*²/df**	4.504	poor	1.381	good	1.083	good	1.432	good	1.111	good
***p*-value**	0	poor	0.087	good	0.355	good	0.068	good	0.337	good
**CFI**	0.086	poor	0.928	poor	1	good	0.92	poor	0.999	good
**RMSEA**	0.124	poor	0.037	good	0	good	0.04	good	0.005	good
**BIC**	8699	5	8616	1	8629	3	8621	2	8660	4
**Heywood**	0	no	0	no	0	no	0	no	3	yes
**YK-N17-300**										
***X*²/df**	4.397	poor	1.232	good	0.944	good	1.27	good	0.88	good
***p*-value**	0	poor	0.185	good	0.537	good	0.158	good	0.593	good
**CFI**	0.097	poor	0.965	accept	1	good	0.96	accept	1	good
**RMSEA**	0.122	poor	0.025	good	0	good	0.028	good	0	good
**BIC**	8700	5	8615	1	8629	3	8620	2	8660	4
**Heywood**	0	no	0	no	0	no	0	no	3	yes

Abbreviations: Est = estimate; Int = interpretation based on Schermelleh-Engel et al. (2003); A55 = Atlas55+; YK = Yeo-Krienen atlas; CFI = comparative fit index, RMSEA = root mean square error of approximation; BIC = Bayesian information criterion.

Note. Grey cells indicated the selected model for each atlas and/or variant. Heywood indicates the number of statistically impossible estimates in the output, such as negative variances or correlations higher 1 or lower -1.

Supplementary Table 4

Summary of Estimates, Standard Error, P-Values, and Confidence Intervals, and for Selected Models for each Atlas and Variant Used

**Medial Frontoparietal Network**						
**Atlas**	**Parameter**	**Estimate**	**SE**	**p-value**	**CI lower**	**CI upper**
A55-N5	Intercept	51.49	0.812	0	49.899	53.08
	Linear Slope	0.139	0.136	0.305	-0.127	0.405
	Variance Intercept	35.838	4.837	0	26.359	45.318
	Residual Variance	77.95	5.807	0	66.569	89.331
	Age on Intercept	-0.198	0.11	0.073	-0.414	0.018
	Sex on Intercept	-3.025	1.047	0.004	-5.076	-0.973
	Education on Intercept	-0.238	0.605	0.694	-1.424	0.948
A55-N15	Intercept	52.003	0.758	0	50.518	53.488
	Linear Slope	-0.28	0.111	0.012	-0.497	-0.062
	Variance Intercept	40.988	5.527	0	30.156	51.82
	Residual Variance	57.134	3.984	0	49.325	64.944
	Age on Intercept	-0.339	0.095	0	-0.526	-0.152
	Sex on Intercept	-3.279	1.024	0.001	-5.285	-1.272
	Education on Intercept	0.401	0.616	0.514	-0.805	1.608
YK-N7-100	Intercept	51.228	0.756	0	49.745	52.71
	Linear Slope	0.042	0.128	0.74	-0.208	0.293
	Variance Intercept	32.672	4.689	0	23.482	41.861
	Residual Variance	63.576	3.539	0	56.639	70.513
	Age on Intercept	-0.321	0.097	0.001	-0.512	-0.131
	Sex on Intercept	-2.53	0.967	0.009	-4.426	-0.634
	Education on Intercept	-0.132	0.582	0.821	-1.272	1.008
YK-N7-200	Intercept	52.352	0.793	0	50.798	53.905
	Linear Slope	-0.255	0.126	0.044	-0.502	-0.007
	Variance Intercept	39.05	5.526	0	28.219	49.881
	Residual Variance	67.929	4.454	0	59.199	76.658

	Age on Intercept	-0.342	0.103	0.001	-0.544	-0.14
	Sex on Intercept	-4.369	1.016	0	-6.359	-2.378
	Education on Intercept	-0.136	0.624	0.828	-1.359	1.087
YK-N7-400	Intercept	51.664	0.777	0	50.14	53.188
	Linear Slope	-0.104	0.127	0.411	-0.353	0.145
	Variance Intercept	36.09	5.362	0	25.58	46.6
	Residual Variance	64.179	3.864	0	56.606	71.752
	Age on Intercept	-0.266	0.099	0.007	-0.46	-0.072
	Sex on Intercept	-2.894	0.976	0.003	-4.806	-0.982
	Education on Intercept	-0.237	0.605	0.695	-1.423	0.948
YK-N17-300	Intercept	52.04	0.777	0	50.517	53.563
	Linear Slope	-0.366	0.117	0.002	-0.596	-0.136
	Variance Intercept	38.114	5.003	0	28.308	47.92
	Residual Variance	63.408	4.819	0	53.964	72.852
	Age on Intercept	-0.338	0.099	0.001	-0.532	-0.145
	Sex on Intercept	-3.348	1.004	0.001	-5.315	-1.38
	Education on Intercept	-0.33	0.617	0.593	-1.54	0.88

Lateral Frontoparietal Network

**Atlas**	**Parameter**	**Estimate**	**SE**	**p-value**	**CI lower**	**CI upper**
A55-N5	Intercept	50.537	0.75	0	49.067	52.008
	Linear Slope	0.798	0.436	0.067	-0.056	1.653
	Quadratic Slope	-0.166	0.063	0.008	-0.288	-0.043
	Variance Intercept	24.048	4.286	0	15.648	32.448
	Residual Variance	76.007	5.71	0	64.815	87.199
	Age on Intercept	-0.031	0.095	0.746	-0.217	0.156
	Sex on Intercept	-1.481	0.952	0.12	-3.348	0.385
	Education on Intercept	-0.106	0.592	0.858	-1.267	1.055
A55-N15	Intercept	51.216	0.864	0	49.523	52.91
	Linear Slope	0.743	0.469	0.114	-0.177	1.662
	Quadratic Slope	-0.166	0.067	0.014	-0.297	-0.034

	Variance Intercept	33.182	9.103	0	15.341	51.023
	Residual Variance	90.047	7.119	0	76.095	103.999
	Age on Intercept	-0.049	0.108	0.653	-0.261	0.163
	Sex on Intercept	-1.69	1.095	0.123	-3.837	0.456
	Education on Intercept	0.54	0.646	0.403	-0.725	1.806
YK-N7-100	Intercept	48.275	0.654	0	46.993	49.557
	Linear Slope	0.853	0.439	0.052	-0.008	1.714
	Quadratic Slope	-0.115	0.062	0.063	-0.236	0.006
	Variance Intercept	17.605	3.464	0	10.817	24.394
	Residual Variance	69.706	3.932	0	62	77.412
	Age on Intercept	0.07	0.077	0.363	-0.081	0.222
	Sex on Intercept	2.947	0.81	0	1.359	4.536
	Education on Intercept	-0.435	0.477	0.362	-1.371	0.5
YK-N7-200	Intercept	49.893	0.648	0	48.622	51.164
	Linear Slope	0.212	0.138	0.124	-0.058	0.482
	Variance Intercept	20.358	3.875	0	12.763	27.953
	Residual Variance	73.253	4.154	0	65.111	81.396
	Age on Intercept	-0.037	0.088	0.675	-0.209	0.135
	Sex on Intercept	1.878	0.869	0.031	0.174	3.582
	Education on Intercept	-0.564	0.495	0.254	-1.533	0.406
YK-N7-400	Intercept	50.315	0.669	0	49.004	51.627
	Linear Slope	0.02	0.14	0.885	-0.255	0.295
	Variance Intercept	21.918	4.238	0	13.611	30.224
	Residual Variance	71.222	4.229	0	62.933	79.511
	Age on Intercept	-0.058	0.093	0.531	-0.24	0.124
	Sex on Intercept	1.1	0.871	0.207	-0.607	2.808
	Education on Intercept	-0.442	0.479	0.356	-1.381	0.496
YK-N17-300	Intercept	50.286	0.698	0	48.919	51.653
	Linear Slope	-0.073	0.141	0.606	-0.35	0.204
	Variance Intercept	19.643	3.885	0	12.028	27.258
	Residual Variance	87.902	10.499	0	67.324	108.481

	Age on Intercept	-0.015	0.092	0.869	-0.195	0.165
	Sex on Intercept	1.211	0.898	0.178	-0.549	2.971
	Education on Intercept	-0.462	0.538	0.391	-1.516	0.593

Midcingulo-Insular Network

**Atlas**	**Parameter**	**Estimate**	**SE**	**p-value**	**CI lower**	**CI upper**
A55-N5	Intercept	49.313	0.651	0	48.037	50.588
	Linear Slope	0.137	0.134	0.307	-0.126	0.4
	Variance Intercept	32.981	8.438	0	16.443	49.52
	Covariance Linear Slope - Quadratic Slope	NA	0	NA	NA	NA
	Residual Variance	65.427	4.455	0	56.695	74.16
	Age on Intercept	-0.021	0.112	0.854	-0.24	0.199
	Sex on Intercept	1.919	0.951	0.044	0.054	3.783
	Education on Intercept	-0.072	0.565	0.898	-1.18	1.036
A55-N15	Intercept	51.398	0.789	0	49.851	52.944
	Linear Slope	-0.281	0.103	0.007	-0.484	-0.079
	Variance Intercept	40.75	8.388	0	24.309	57.19
	Residual Variance	54.863	3.754	0	47.505	62.22
	Age on Intercept	-0.16	0.108	0.138	-0.371	0.052
	Sex on Intercept	-1.718	0.995	0.084	-3.668	0.232
	Education on Intercept	0.17	0.637	0.79	-1.079	1.419
YK-N7-100	Intercept	49.954	0.745	0	48.493	51.414
	Linear Slope	0.591	0.435	0.174	-0.262	1.443
	Quadratic Slope	-0.177	0.062	0.004	-0.298	-0.056
	Variance Intercept	32.205	5.726	0	20.982	43.427
	Residual Variance	68.089	4.413	0	59.439	76.739
	Age on Intercept	-0.364	0.106	0.001	-0.572	-0.157
	Sex on Intercept	-0.333	0.974	0.732	-2.243	1.577
	Education on Intercept	0.333	0.541	0.538	-0.727	1.393
	Intercept	50.009	0.761	0	48.517	51.501

YK-N7-200	Linear Slope	0.75	0.428	0.08	-0.09	1.589
	Quadratic Slope	-0.179	0.06	0.003	-0.297	-0.061
	Variance Intercept	37.585	5.627	0	26.556	48.615
	Residual Variance	63.059	4.293	0	54.645	71.474
	Age on Intercept	-0.321	0.1	0.001	-0.518	-0.124
	Sex on Intercept	-0.2	0.994	0.84	-2.148	1.747
	Education on Intercept	0.145	0.585	0.805	-1.002	1.291
YK-N7-400	Intercept	50.242	0.753	0	48.766	51.718
	Linear Slope	0.887	0.429	0.039	0.047	1.727
	Quadratic Slope	-0.187	0.061	0.002	-0.306	-0.067
	Variance Intercept	35.16	5.312	0	24.75	45.571
	Variance Linear Slope	NA	0	NA	NA	NA
	Residual Variance	62.093	3.918	0	54.413	69.772
	Age on Intercept	-0.331	0.1	0.001	-0.527	-0.134
	Sex on Intercept	-0.356	0.984	0.717	-2.284	1.572
	Education on Intercept	0.184	0.566	0.745	-0.925	1.292
YK-N17-300	Intercept	50.236	0.73	0	48.806	51.666
	Linear Slope	0.902	0.432	0.037	0.054	1.749
	Quadratic Slope	-0.19	0.063	0.003	-0.313	-0.066
	Variance Intercept	33.532	5.782	0	22.199	44.866
	Residual Variance	71.471	6.64	0	58.457	84.485
	Age on Intercept	-0.314	0.109	0.004	-0.526	-0.101
	Sex on Intercept	-0.323	0.979	0.741	-2.241	1.595
	Education on Intercept	0.215	0.574	0.707	-0.91	1.341

Pericentral Network

**Atlas**	**Parameter**	**Estimate**	**SE**	**p-value**	**CI lower**	**CI upper**
A55-N5	Intercept	47.604	0.826	0	45.985	49.222
	Linear Slope	-0.276	0.595	0.643	-1.442	0.89
	Quadratic Slope	0.076	0.082	0.357	-0.085	0.237
	Variance Intercept	38.138	8.585	0	21.311	54.965

	Variance Linear Slope	10.415	6.051	0.085	-1.445	22.275
	Variance Quadratic Slope	0.188	0.124	0.131	-0.056	0.432
	Covariance Intercept - Linear Slope	-9.989	6.014	0.097	-21.777	1.798
	Covariance Intercept - Quadratic Slope	1.55	0.856	0.07	-0.127	3.227
	Covariance Linear Slope - Quadratic Slope	-1.398	0.862	0.105	-3.087	0.291
	Residual Variance	61.859	5.318	0	51.437	72.282
	Age on Intercept	-0.115	0.135	0.393	-0.38	0.149
	Sex on Intercept	4.445	1.268	0	1.96	6.929
	Education on Intercept	-0.194	0.753	0.797	-1.669	1.281
	Age on Linear Slope	0.01	0.104	0.923	-0.193	0.214
	Sex on Linear Slope	1.144	0.891	0.199	-0.602	2.89
	Education on Linear Slope	-0.418	0.556	0.452	-1.507	0.671
	Age on Quadratic Slope	-0.003	0.014	0.823	-0.031	0.025
	Sex on Quadratic Slope	-0.236	0.123	0.056	-0.478	0.006
	Education on Quadratic Slope	0.063	0.076	0.409	-0.086	0.211
A55-N15	Intercept	47.06	0.71	0	45.669	48.451
	Linear Slope	1.064	0.431	0.014	0.219	1.909
	Quadratic Slope	-0.172	0.062	0.006	-0.294	-0.049
	Variance Intercept	36.396	5.648	0	25.326	47.466
	Residual Variance	63.599	4.154	0	55.459	71.74
	Age on Intercept	-0.165	0.094	0.079	-0.349	0.019
	Sex on Intercept	5.47	0.988	0	3.534	7.405
	Education on Intercept	-0.487	0.57	0.393	-1.603	0.63
YK-N7-100	Intercept	47.862	0.716	0	46.46	49.265
	Linear Slope	0.904	0.418	0.031	0.084	1.723
	Quadratic Slope	-0.153	0.06	0.01	-0.269	-0.036
	Variance Intercept	31.998	5.069	0	22.064	41.932
	Residual Variance	66.416	4.431	0	57.732	75.099

	Age on Intercept	-0.408	0.085	0	-0.575	-0.24
	Sex on Intercept	4.516	0.967	0	2.621	6.411
	Education on Intercept	-0.382	0.538	0.478	-1.436	0.673
YK-N7-200	Intercept	47.49	0.728	0	46.062	48.917
	Linear Slope	0.929	0.404	0.022	0.136	1.722
	Quadratic Slope	-0.143	0.057	0.012	-0.255	-0.031
	Variance Intercept	36.636	5.583	0	25.692	47.579
	Residual Variance	61.248	4.186	0	53.044	69.452
	Age on Intercept	-0.299	0.09	0.001	-0.476	-0.121
	Sex on Intercept	5.401	0.978	0	3.485	7.317
	Education on Intercept	-0.379	0.544	0.486	-1.445	0.687
YK-N7-400	Intercept	47.144	0.715	0	45.743	48.546
	Linear Slope	1.252	0.394	0.001	0.48	2.024
	Quadratic Slope	-0.17	0.056	0.002	-0.28	-0.06
	Variance Intercept	39.393	5.704	0	28.213	50.572
	Residual Variance	59.099	4.282	0	50.706	67.492
	Age on Intercept	-0.27	0.09	0.003	-0.447	-0.094
	Sex on Intercept	6.101	1.001	0	4.139	8.063
	Education on Intercept	-0.412	0.56	0.462	-1.51	0.685
YK-N17-300	Intercept	47.367	0.706	0	45.983	48.751
	Linear Slope	1.091	0.394	0.006	0.319	1.864
	Quadratic Slope	-0.161	0.056	0.004	-0.271	-0.051
	Variance Intercept	36.898	5.708	0	25.712	48.085
	Residual Variance	60.718	4.38	0	52.135	69.302
	Age on Intercept	-0.304	0.088	0.001	-0.478	-0.131
	Sex on Intercept	5.818	0.991	0	3.876	7.76
	Education on Intercept	-0.477	0.553	0.388	-1.56	0.606

Occipital Network

Atlas	Parameter	Estimate	SE	p-value	CI lower	CI upper
A55-N5	Intercept	48.946	0.747	0	47.482	50.41

	Linear Slope	0.236	0.205	0.25	-0.166	0.638
	Variance Intercept	32.63	7.409	0	18.109	47.15
	Variance Linear Slope	0.636	0.345	0.065	-0.04	1.311
	Covariance Intercept - Linear Slope	-2.843	1.266	0.025	-5.324	-0.362
	Residual Variance	55.036	4.015	0	47.166	62.906
	Age on Intercept	0.002	0.111	0.983	-0.215	0.22
	Sex on Intercept	0.161	1.063	0.879	-1.921	2.244
	Education on Intercept	-0.526	0.593	0.375	-1.689	0.636
	Age on Linear Slope	0.005	0.03	0.873	-0.054	0.064
	Sex on Linear Slope	-0.483	0.269	0.073	-1.009	0.044
	Education on Linear Slope	0.262	0.159	0.099	-0.05	0.573
A55-N15	Intercept	49.174	0.783	0	47.641	50.708
	Linear Slope	0.565	0.232	0.015	0.111	1.019
	Variance Intercept	38.132	8.346	0	21.775	54.489
	Variance Linear Slope	1.069	0.418	0.011	0.25	1.888
	Covariance Intercept - Linear Slope	-3.041	1.516	0.045	-6.011	-0.071
	Residual Variance	60.328	4.483	0	51.542	69.115
	Age on Intercept	0.069	0.117	0.555	-0.161	0.299
	Sex on Intercept	0.472	1.182	0.69	-1.846	2.789
	Education on Intercept	-0.204	0.646	0.753	-1.469	1.062
	Age on Linear Slope	0.011	0.033	0.733	-0.053	0.076
	Sex on Linear Slope	-0.58	0.313	0.063	-1.193	0.032
	Education on Linear Slope	0.19	0.19	0.316	-0.182	0.562
YK-N7-100	Intercept	48.717	0.786	0	47.176	50.258
	Linear Slope	0.395	0.214	0.065	-0.025	0.815
	Variance Intercept	33.571	7.012	0	19.828	47.313
	Variance Linear Slope	1.124	0.454	0.013	0.234	2.014
	Covariance Intercept - Linear Slope	-2.511	1.311	0.055	-5.08	0.057
	Residual Variance	56.781	4.052	0	48.838	64.723

	Age on Intercept	0.176	0.114	0.124	-0.048	0.399
	Sex on Intercept	0.985	1.083	0.363	-1.138	3.108
	Education on Intercept	0.098	0.608	0.872	-1.093	1.289
	Age on Linear Slope	-0.022	0.031	0.47	-0.083	0.038
	Sex on Linear Slope	-0.605	0.301	0.045	-1.196	-0.015
	Education on Linear Slope	0.105	0.172	0.54	-0.232	0.443
YK-N7-200	Intercept	49.143	0.806	0	47.564	50.722
	Linear Slope	0.448	0.208	0.031	0.04	0.856
	Variance Intercept	35.342	8.268	0	19.136	51.547
	Variance Linear Slope	0.772	0.404	0.056	-0.02	1.564
	Covariance Intercept - Linear Slope	-1.844	1.333	0.166	-4.456	0.768
	Residual Variance	59.404	4.377	0	50.825	67.983
	Age on Intercept	0.153	0.114	0.18	-0.071	0.377
	Sex on Intercept	1.013	1.121	0.366	-1.184	3.211
	Education on Intercept	-0.332	0.636	0.602	-1.58	0.915
	Age on Linear Slope	-0.017	0.029	0.554	-0.075	0.04
	Sex on Linear Slope	-0.693	0.293	0.018	-1.268	-0.118
	Education on Linear Slope	0.197	0.169	0.245	-0.135	0.529
YK-N7-400	Intercept	48.848	0.784	0	47.311	50.384
	Linear Slope	0.491	0.212	0.02	0.076	0.907
	Variance Intercept	35.94	7.792	0	20.667	51.212
	Variance Linear Slope	0.834	0.389	0.032	0.072	1.596
	Covariance Intercept - Linear Slope	-1.89	1.237	0.126	-4.314	0.534
	Residual Variance	58.334	4.151	0	50.198	66.47
	Age on Intercept	0.149	0.119	0.21	-0.084	0.382
	Sex on Intercept	1.743	1.124	0.121	-0.46	3.946
	Education on Intercept	-0.562	0.638	0.378	-1.812	0.688
	Age on Linear Slope	-0.017	0.03	0.581	-0.076	0.043
	Sex on Linear Slope	-0.687	0.296	0.02	-1.267	-0.108

	Education on Linear Slope	0.238	0.171	0.165	-0.098	0.573
YK-N17-300	Intercept	49.232	0.796	0	47.672	50.791
	Linear Slope	0.448	0.205	0.029	0.046	0.85
	Variance Intercept	37.879	9.285	0	19.681	56.077
	Variance Linear Slope	0.799	0.371	0.031	0.072	1.525
	Covariance Intercept - Linear Slope	-2.216	1.41	0.116	-4.979	0.546
	Residual Variance	58.368	4.632	0	49.289	67.448
	Age on Intercept	0.11	0.12	0.361	-0.126	0.346
	Sex on Intercept	1.149	1.144	0.315	-1.094	3.392
	Education on Intercept	-0.321	0.646	0.62	-1.587	0.946
	Age on Linear Slope	-0.005	0.03	0.856	-0.063	0.053
	Sex on Linear Slope	-0.684	0.289	0.018	-1.25	-0.119
	Education on Linear Slope	0.196	0.168	0.243	-0.133	0.524

Abbreviations: A55 = Atlas55+; YK = Yeo-Krienen atlas.

Supplementary Table 5

Median and Range of Regional Homogeneity and Silhouette Coefficient for each Network and Atlas.

	**Regional Homogeneity – Median (Range)**					
	**A55_N5**	**A55_N15**	**YK_N7_100**	**YK_N7_200**	**YK_N7_400**	**YK_N17_300**
**ON**	0.25 (0.21; 0.31)	0.26 (0.20; 0.32)	0.25 (0.21; 0.31)	0.25 (0.21; 0.32)	0.25 (0.21; 0.31)	0.25 (0.20; 0.32)
**PN**	0.24 (0.21; 0.31)	0.24 (0.20; 0.31)	0.24 (0.20; 0.31)	0.24 (0.20; 0.31)	0.24 (0.20; 0.31)	0.24 (0.20; 0.31)
**M-FPN**	0.25 (0.21; 0.30)	0.25 (0.21; 0.31)	0.27 (0.22; 0.32)	0.27 (0.22; 0.32)	0.27 (0.22; 0.33)	0.27 (0.22; 0.32)
**L-FPN**	0.25 (0.22; 0.30)	0.26 (0.22; 0.31)	0.28 (0.23; 0.33)	0.26 (0.21; 0.31)	0.27 (0.22; 0.31)	0.27 (0.23; 0.32)
**M-CIN**	0.24 (0.20; 0.29)	0.25 (0.21; 0.30)	0.26 (0.22; 0.31)	0.26 (0.22; 0.31)	0.26 (0.22; 0.31)	0.26 (0.22; 0.31)

	Silhouette Coefficient – Median (Range)					
	**A55_N5**	**A55_N15**	**YK_N7_100**	**YK_N7_200**	**YK_N7_400**	**YK_N17_300**
**ON**	0.10 (-0.01; 0.33)	0.14 (0.00; 0.54)	0.16 (-0.05; 0.42)	0.13 (0.00; 0.39)	0.09 (0.00; 0.28)	0.14 (0.01; 0.41)
**PN**	0.05 (-0.01; 0.18)	0.02 (-0.04; 0.18)	0.07 (-0.05; 0.39)	0.03 (-0.04; 0.25)	0.01 (-0.03; 0.17)	0.03 (-0.03; 0.24)
**M-FPN**	-0.01 (-0.05; 0.05)	0.02 (-0.03; 0.13)	-0.01 (-0.09; 0.11)	0.01 (-0.05; 0.08)	0.01 (-0.04; 0.09)	0.02 (-0.03; 0.11)
**L-FPN**	-0.02 (-0.06; 0.03)	-0.03 (-0.06; 0.01)	0.01 (-0.12; 0.18)	-0.01 (-0.08; 0.06)	0.00 (-0.05; 0.06)	-0.01 (-0.06; 0.05)
**M-CIN**	-0.03 (-0.09; 0.01)	0.01 (-0.07; 0.08)	0.04 (-0.06; 0.29)	0.03 (-0.05; 0.19)	0.02 (-0.03; 0.16)	0.01 (-0.05; 0.19)

Abbreviations: A55 = Atlas55+; YK = Yeo-Krienen atlas; ON = Occipital Network; PN = Pericentral Network; M-FPN = Meidal Frontoparietal Network; L-FPN = Lateral Frontoparietal Network; M-CIN = Midcingulo-Insular Network

Supplementary Table 6

Spearman Rank Correlation Coefficient and Adjusted P-Value for Associations of Age and Regional Homogeneity / Silhouette Coefficient for each Network and Atlas.

	Regional Homogeneity – Age Correlation and Adjusted *P*-Value					
	**A55_N5**	**A55_N15**	**YK_N7_100**	**YK_N7_200**	**YK_N7_400**	**YK_N17_300**
**ON**	*r* = 0.132, *p* = 0.346	*r* = 0.117, *p* = 0.557	*r* = 0.136, *p* = 0.297	*r* = 0.134, *p* = 0.323	*r* = 0.131, *p* = 0.354	*r* = 0.139, *p* = 0.269
**PN**	*r* = 0.156, *p* = 0.144	*r* = 0.178, *p* = 0.060	*r* = 0.140, *p* = 0.256	*r* = 0.176, *p* = 0.064	*r* = 0.169, *p* = 0.087	*r* = 0.173, *p* = 0.074
**M-FPN**	***r* = 0.184, *p* = 0.046**	*r* = 0.145, *p* = 0.217	*r* = 0.116, *p* = 0.567	*r* = 0.117, *p* = 0.558	*r* = 0.117, *p* = 0.550	*r* = 0.116, *p* = 0.573

**L-FPN**	$r = 0.125$, $p = 0.432$	$r = 0.141$, $p = 0.252$	$r = 0.131$, $p = 0.355$	$r = 0.155$, $p = 0.152$	$r = 0.147$, $p = 0.204$	$r = 0.181$, $p = 0.053$
**M-CIN**	$r = 0.166$, $p = 0.098$	$r = 0.107$, $p = 0.740$	$r = 0.155$, $p = 0.153$	$r = 0.158$, $p = 0.136$	$r = 0.164$, $p = 0.108$	$r = 0.146$, $p = 0.210$
	**Silhouette Coefficient – Age Correlation and Adjusted *P*-Value**					
	**A55_N5**	**A55_N15**	**YK_N7_100**	**YK_N7_200**	**YK_N7_400**	**YK_N17_300**
**ON**	$r = 0.066$, $p = 1.000$	$r = 0.052$, $p = 1.000$	$r = 0.041$, $p = 1.000$	$r = 0.051$, $p = 1.000$	$r = 0.078$, $p = 1.000$	$r = 0.073$, $p = 1.000$
**PN**	$r = -0.046$, $p = 1.000$	$r = -0.042$, $p = 1.000$	$r = -0.005$, $p = 0.578$	$r = -0.001$, $p = 1.000$	$r = -0.021$, $p = 1.000$	$r = -0.022$, $p = 1.000$
**M-FPN**	$r = -0.025$, $p = 1.000$	$r = -0.006$, $p = 1.000$	$r = 0.091$, $p = 1.000$	$r = 0.068$, $p = 1.000$	$r = 0.042$, $p = 1.000$	$r = 0.017$, $p = 1.000$
**L-FPN**	$r = -0.112$, $p = 0.649$	$r = -0.110$, $p = 0.674$	$r = -0.008$, $p = 1.000$	$r = 0.003$, $p = 1.000$	$r = -0.037$, $p = 1.000$	$r = -0.033$, $p = 1.000$
**M-CIN**	$r = -0.026$, $p = 1.000$	$r = -0.012$, $p = 1.000$	$r = -0.016$, $p = 1.000$	$r = -0.002$, $p = 1.000$	$r = -0.004$, $p = 1.000$	$r = -0.021$, $p = 1.000$

Abbreviations: A55 = Atlas55+; YK = Yeo-Krienen atlas; ON = Occipital Network; PN = Pericentral Network; M-FPN = Meidal Frontoparietal Network; L-FPN = Lateral Frontoparietal Network; M-CIN = Midcingulo-Insular Network

Supplementary Table 7

Median and Range of Regional Homogeneity and Silhouette Coefficient for each Network of the A55-N5 and A55-N15 as Defined with Nodes by Schaefer.

	**Regional Homogeneity**		**Silhouette Coefficient**	
**Networks**	**A55-N5-400**	**A55-N15-300**	**A55-N5-400**	**A55-N15-300**
**ON**	0.25 (0.21; 0.32)	0.26 (0.21; 0.33)	0.10 (0.01; 0.33)	0.12 (0.02; 0.37)
**PN**	0.24 (0.20; 0.31)	0.24 (0.20; 0.31)	0.01 (-0.04; 0.14)	0.00 (-0.04; 0.14)
**M-FPN**	0.25 (0.21; 0.31)	0.25 (0.21; 0.30)	0.00 (-0.04; 0.05)	0.01 (-0.04; 0.07)
**L-FPN**	0.26 (0.22; 0.30)	0.26 (0.22; 0.31)	-0.01 (-0.05; 0.03)	-0.03 (-0.08; 0.01)
**M-CIN**	0.26 (0.21; 0.31)	0.25 (0.22; 0.30)	-0.01 (-0.04; 0.03)	0.00 (-0.05; 0.11)

Abbreviations: A55 = Atlas55+; ON = Occipital Network; PN = Pericentral Network; M-FPN = Meidal Frontoparietal Network; L-FPN = Lateral Frontoparietal Network; M-CIN = Midcingulo-Insular Network

Note. A55-N5 and A55-N15 correspond to the original Atlas55+ and were analyzed using nodes of the automated anatomical labeling atlas. A55-N5-400 and A55-N15-300 correspond to the networks when assigning the 400 or 300 Schaefers node to the networks by Atlas55+ that show the highest overlap. A more detailed description of the node assignment to the A55 network can be found in the main text post-hoc analysis of atlas fit.

Supplementary Table 8

Dice Similarity Coefficient for each Network between the A55-N5 (original) and A55-N5-400 (nodes by Schaefer) and the A55-N15 and A55-N15-300.

	**ON**	**PN**	**M-FPN**	**L-FPN**	**M-CIN**
**A55-N5 vs. A55-N5-400**	0.797	0.761	0.650	0.639	0.595
**A55-N15 vs. A55-N15-300**	0.740	0.678	0.545	0.547	0.337

Abbreviations: A55 = Atlas55+; ON = Occipital Network; PN = Pericentral Network; M-FPN = Meidal Frontoparietal Network; L-FPN = Lateral Frontoparietal Network; M-CIN = Midcingulo-Insular Network

Note. A55-N5 and A55-N15 correspond to the original Atlas55+ and were analyzed using nodes of the automated anatomical labeling atlas. A55-N5-400 and A55-N15-300 correspond to the networks when assigning the 400 or 300 Schaefers node to the networks by Atlas55+ that show the highest overlap. A more detailed description of the node assignment to the A55 network can be found in the main text post-hoc analysis of atlas fit.
